# The Importance of Going beyond the Independent Atom Model When Predicting UED Signals from Simulations

**DOI:** 10.1021/acs.jctc.6c00207

**Published:** 2026-06-10

**Authors:** Lewis Hutton, Andrés Moreno Carrascosa, Mats Simmermacher, Adam Kirrander

**Affiliations:** Physical and Theoretical Chemistry Laboratory, Department of Chemistry, 6396 University of Oxford, Oxford OX1 3QZ, U.K.

## Abstract

The level of theory required to predict ultrafast electron diffraction signals is investigated in photoexcited γ-butyrolactone. The total isotropic diffraction signal is calculated both with the independent atom model (IAM) and directly from *ab initio* electronic wave functions. The results are benchmarked at the equilibrium geometry, along a representative photochemical reaction coordinate (for both ground and excited electronic states), and, most importantly, for a full fewest-switches surface-hopping simulation of the nonadiabatic dynamics. The IAM qualitatively captures the time-resolved UED signal, but there are deviations between the IAM and the *ab initio* results, irrespective of the electronic structure method used. The errors of the IAM do not get washed out even when the nuclear wavepacket is considered. The errors primarily manifest in the intensity rather than position of scattering features, particularly at small to medium values of momentum transfer, and are sufficient to affect lifetimes and other parameters determined from the diffraction signal. Our results indicate that the IAM can lead to errors when scattering signals are inverted. Crucially, calculations beyond the IAM are necessary to account for the effects of chemical bonding, charge transfer, electronic excitation and ionization.

## Graphical abstract

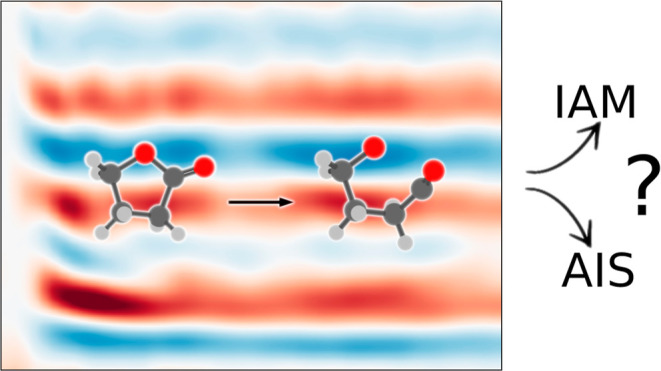

## 1 Introduction

Ultrafast electron diffraction (UED) has emerged as a powerful technique for studying chemical dynamics.^1–3^ Together with the closely related ultrafast X-ray scattering (UXS), it has been shown to provide ready access to the structural dynamics of molecules.^4–10^ The experiments are often interpreted using the independent atom model (IAM),^11–14^ whose conceptual and computational simplicity has made it an accessible go-to tool for predicting experimental signals. An example of this is the 2025 “Cyclobutanone Prediction Challenge” ^15–30^ where a number of theory groups attempted to predict the UED signal for a yet unpublished experiment, with the IAM used almost exclusively. Two groups included analysis of beyond-IAM scattering in their Supporting Information.^25,30^ Equally, most published UED experiments rely on the IAM for their analysis.

Given the widespread and persistent use of the IAM, a meticulous examination of its accuracy is essential. The timeliness of this examination is further underscored by recent scattering experiments that resolve electronic effects, for instance associated with excited electronic states, which cannot be described by the IAM.^6,31–36^

This paper sets out to quantify the shortcomings of the IAM in the context of time-dependent nonadiabatic dynamics, using simulations of the photoexcited dynamics of the molecule γ-butyrolactone as a representative test-case.^37–40^ This benchmark and quantitative analysis of the performance of IAM is made possible by comparison to state-of-the-art calculations of the scattering signals from *ab initio* electronic wave functions.^41–43^ The comparison is done at the static equilibrium geometry and along a representative reaction coordinate, looking at both ground and excited electronic states. We then examine the differences between the IAM and *ab initio* scattering (AIS) for the time-dependent signal. This allows us to systematically quantify the differences between the IAM and AIS, examine how the level of electronic-structure theory used to calculate the wave function affects the scattering signal, and draw qualitative and quantitative conclusions. Finally, we investigate how the method used to predict the UED signal affects secondary properties such as life times determined from the observable.

## 2 Theory

The two options for calculating electron or X-ray scattering are the widely used independent atom model (IAM) and *ab initio* scattering (AIS).

### 2.1 Independent Atom Model

The IAM approximates molecular scattering as a coherent sum of scattering amplitudes from independent atoms, using tabulated atomic form factors.^44^ The elastic form factors are *f*_α_^ e^(*s*), with *s* = | ***k***_0_ – ***k***_1_| being the norm of the momentum transfer vector defined using the incoming ***k***_0_ and outgoing ***k***_1_ wavevectors. The inelastic part is added incoherently via Compton factors *S*_α_^inel^(*s*).^44^ The elastic form factors for electron scattering must account for scattering from the atomic electrons and the nucleus, and are hence given by *f*_α_^ e^(*s*) = *f*_α_^ x^(*s*) – *Z*_α_, where *f*_α_^ x^(*s*) are the X-ray scattering form factors and *Z*_α_ the nuclear charge of the atom.^44^ The total (energy-integrated) scattering $I_{IAM}\left(s,\mathrm{R̅}\right)$ is then obtained as the sum of elastic and inelastic components. In rotationally averaged form this is^1,10^

1 $$I_{IAM}\left(s,\mathrm{R̅}\right)=\sum_{\alpha }^{N_{at}}|f_{\alpha }^{e}\left(s\right){|}^{2}+2\sum_{\alpha <\beta }^{N_{at}}f_{\alpha }^{e}\left(s\right)f_{\beta }^{e}\left(s\right)\mathrm{sinc}\left(sR_{\alpha \beta }\right)+\sum_{\alpha }^{N_{at}}S_{\alpha }^{inel}\left(s\right)$$

in eq 1, and throughout this paper, the scattering is considered in units of the Rutherford cross section (dσ/dΩ)_Ru_.^10^ The indices α and β in eq 1 run over the *N*_at_ atoms of the molecule, with the internuclear distances *R*_αβ_ = | ***R***_β_ – ***R***_α_| calculated using the nuclear coordinate vectors ***R***_α_, obtained from the molecular geometry $\mathrm{R̅}=\left(R_{1},...,R_{N_{at}}\right)$ .

### 2.2 *Ab initio* Scattering

The IAM, however, has several well-known shortcomings, leading to an inadequate description of the electron density, an inability to account for the changes in electron density between different electronic states, and an inelastic component that does not account for the molecule’s electronic structure and geometry.^41,42,45^ Overcoming these shortcomings involves calculating the scattering from the electronic wave function. A number of methods to calculate electron and X-ray scattering from first-principles have been developed, making it possible to evaluate elastic, inelastic, coherent mixed, and total differential cross sections.^41,43,45–63^

In brief, the *ab initio* total scattering signal for a specific state *a* is given by^10^

$$I_{AIS}\left(a,s,\mathrm{R̅}\right)=\langle \Psi_{a}\left(\mathrm{R̅}\right)|{Ẑ}^{†}\left(s\right)Ẑ\left(s\right)|\Psi_{a}{\left(\mathrm{R̅}\right)\rangle }_{0}$$ 2

where $\Psi_{a}\left(\mathrm{r̅};\mathrm{R̅}\right)=\left\langle \mathrm{r̅}|\Psi_{a}\left(\mathrm{R̅}\right)\right\rangle$ is the wave function of electronic state *a* that depends on the coordinates of the *N*_e_ electrons, $\mathrm{r̅}=\left\{r_{1},...,r_{N_{e}}\right\}$ , and parametrically on the coordinates of the *N*_at_ nuclei, $\mathrm{R̅}=\left\{R_{1},...,R_{N_{at}}\right\}$ . Moreover, Ẑ(s) is the electron scattering operator

3 $$Ẑ\left(s\right)=\sum_{i=1}^{N_{e}}e^{ısr_{i}}-\sum_{\alpha =1}^{N_{at}}Z_{\alpha }e^{ısR_{\alpha }}$$

where the first term on the right-hand-side is equal to the X-ray scattering operator *L̂*(*s*). In eq 2, the subscript 0 shows that we consider the isotropic component of the scattering signal. Notably, we ignore the possibility of coherent mixed scattering due to transient electronic wavepackets, which would involve cross-terms between different electronic states.^50,54^ Examining the matrix element in eq 2 and the electron scattering operator in eq 3, we can deduce that the scattering signal has three contributions: one from the nuclei, one from the electrons, and a cross-term between electrons and nuclei. For further information, see e.g. ref 10.

### 2.3 Time Evolution of the Signal

To calculate a time-dependent scattering signal according to eqs 1 or 2 above, the time-evolution of the molecular wave function must be known.^64,65^ Ideally, this should be obtained by propagating the molecular wave function from the initial stationary state using the full time-dependent Schrödinger equation.^54^ However, for molecules beyond two or three atoms, this is a challenging proposition and we resort to approximations. In many cases, trajectory-based on-the-fly methods that propagate the nuclear dynamics (semi)­classically or quantum mechanically are chosen, such as *ab initio* multiple spawning (AIMS),^66–68^ multiconfigurational Ehrenfest (MCE),^69^ direct dynamics variational multiconfigurational Gaussians (dd-vMCG),^70,71^ and trajectory surface hopping (TSH) often with fewest switches surface hopping (FSSH).^72,73^ In the following, we employ FSSH due to its effective compromise between computational cost and accuracy, providing a suitable framework for evaluating the importance of how UED signals are calculated. FSSH is a mixed quantum-classical method that treats the electrons quantum mechanically and the nuclei classically. Discrete transitions (hops) between electronic states account for nonadiabatic behavior and determine the active electronic state *a* for each trajectory. Although a single trajectory is a poor representation of the nuclear wavepacket, an ensemble of trajectories can reproduce correct branching ratios.^72^ Irrespective of the propagation method used, the electronic structure problem must be solved at each step to yield the electronic energies, gradients, and nonadiabatic couplings. These can be calculated using a variety of electronic structure methods, including XMS-CASPT2, SA-CASSCF, ADC(2), and TD-TDDFT.^74–78^ The choice of electronic structure method has a significant impact on the veracity of the dynamics.^79–85^

For trajectory surface hopping simulations (TSH), the overall scattering signal is obtained as an average over the *N*_trj_ trajectories in the ensemble^86,87^

$$I\left(s,t\right)=N_{trj}^{-1}\overset{N_{trj}}{\underset{n}{\sum }}I_{method}\left(a_{n}(t),{s,\mathrm{R̅}}_{n}\left(t\right)\right)$$ 4

where the scattering *I*_method_ is obtained from the IAM (eq 1) or from AIS (eq 2). In the case where scattering is obtained from AIS, the labeling “method” also refers to the electronic structure method by which AIS is calculated. Each trajectory *n* is represented by the time-dependent molecular geometry ${\mathrm{R̅}}_{n}\left(t\right)$ and the active state index *a*_*n*_(*t*) as a function of time *t*. For the IAM in eq 1 the active space index *a*_*n*_ does not affect the results. Note that eq 4 is commensurate with the classical trajectories of TSH and takes a somewhat different form for trajectories based on Gaussian wavepackets such as MCE or AIMS.^64^

## 3 Computational Details

### 3.1 Static Calculations

The ground-state equilibrium structure of γ-butyrolactone was optimized using SA(3)-CASSCF­(10,8)/cc-pVDZ in OpenMolcas V2023.^88^ A summary of the (10,8) active space is provided in the Supporting Information Figure S1, with the other active spaces employed summarized in Table S1. Dunning’s double-ζ correlation consistent Cartesian basis set, cc-pVDZ, is used unless otherwise specified. A Cartesian basis set is required for scattering calculations with our in-house code^42,43,91^ and was therefore used throughout. A Hessian calculation confirmed that the optimized geometry was a true minimum with no imaginary frequencies. Molecular geometries for the S_2_/S_1_ and S_1_/S_0_ minimum-energy conical intersections (MECIs) were optimized also using SA(3)-CASSCF­(10,8)/cc-pVDZ. XMS(3)-CASPT2 calculations were performed with OpenMolcas V2023 ^88^ with the same (10,8) active space and an imaginary shift of 0.5 E_h_. Single-point third-order coupled cluster (CC3) linear-response calculations were performed using the *e* ^*T*^ software^89^ for the three lowest electronic singlet states (S_0_, S_1_ and S_2_) (Table S2) in order to validate the XMS-CASPT2 calculations. Linear interpolation in internal coordinates (LIIC) was used to construct an approximate reaction pathway connecting the equilibrium geometry to the two MECIs, with energies calculated at the SA(3)-CASSCF­(10,8)/cc-pVDZ level in Molpro V2015.1.^90^ The isotropic UED signals were calculated using our in-house code, which evaluates the scattering as a zeroth-order spherical Bessel transform of the two-electron densities from quantum chemical calculations using SA-CASSCF and CCSD.^42,43^ CCSD calculations were performed using the PySCF package.^92^

### 3.2 Nonadiabatic Dynamics Simulations

TSH simulations were performed using SHARC V3.0 in the Molecular Coulomb Hamiltonian (MCH) representation interfaced to OpenMolcas V2023 for the SA(3)-CASSCF­(10,8)/cc-pVDZ electronic structure calculations.^88,93^ Initial conditions in phase-space were sampled from a ground-state harmonic Wigner distribution at 0 K, using the frequencies calculated at the SA(3)-CASSCF­(10,8)/cc-pVDZ level without any energy restriction. We initiated 158 trajectories in the bright S_2_ state and propagated them by FSSH for 200 fs with a time step of 0.2 fs. A local diabatization scheme was used for the propagation and velocities were rescaled along the total velocity vector after hops. An energy-based decoherence scheme was employed with a decoherence parameter of 0.1 E_h_.^94^ After inspection, 36 trajectories were discarded (∼20%) due to lack of energy conservation (further details in the Supporting Information), leaving 122 trajectories for the final analysis.

### 3.3 UED Signals

The static UED signals calculated using each method are presented as percent difference signals

5 $$\%\Delta I_{method}\left(s,\mathrm{R̅}\right)=100\times \frac{I_{method}\left(s,\mathrm{R̅}\right)-I_{ref}\left(s,\mathrm{R̅}\right)}{I_{ref}\left(s,\mathrm{R̅}\right)}$$

where $I_{ref}\left(s,\mathrm{R̅}\right)$ is the reference scattering signal. Similarly, time-resolved UED signals are shown as a percent differences relative to the pump-off signal, *I*_method_ (*s*, *t* < 0)

6 $$\%\Delta I_{method}\left(s,t\right)=100\times \frac{I_{method}\left(s,t\right)-I_{method}\left(s,t<0\right)}{I_{method}\left(s,t<0\right)}$$

where the pump-off signal *I*_method_(*s*, *t* < 0) is calculated on the electronic ground state using the molecular geometries for each of the *N*_trj_ trajectories at *t* = 0. The effect of the sampling of the ground-state equilibrium geometries on the reference scattering signal and hence the percent difference signal is investigated in Figures S2–S4, and indicates that using a single reference geometry is insufficient but that using the initial geometries for the trajectory simulations yields acceptable results. All time-resolved AIS signals were calculated according to eq 2 at the SA(3)-CASSCF­(10,8)/cc-pVDZ level of theory in Molpro V2015.1.^90^ To ensure the (10,8) active space remained consistent between the two sets of calculations (Molpro vs OpenMolcas), we validated the absolute energies against one another and found no discrepancies. CI vectors were also checked between the programs to ensure consistency.

We note that in the sudden approximation, where the S_0_ → S_2_ excitation at *t* = 0 fs is instantaneous, the percent difference signal at *t* = 0 fs is nonzero due to the change of electronic state even though the molecular geometries are the same as in the pump-off reference signal *I*_method_ (*s*, *t* < 0). In contrast, for the IAM, which does not distinguish electronic states, these two signals are identical.

## 4 Results and Discussion

### 4.1 Photochemistry of γ-Butyrolactone

The comparison between IAM and AIS, including evaluating the effect of the active space for the AIS, is done in the molecule γ-butyrolactone. Previous simulations of the photoexcited dynamics of this molecule has shown that it undergoes an ultrafast ring-opening reaction on a sub-50 fs time scale after photoexcitation to the S_2_ state.^37–40^ In the current section, we examine the electronic structure, the key characteristic geometries, and the nonadiabatic dynamics of this molecule, which will inform the analysis of the scattering signals presented in Sections 4.2 and 4.3.

#### 4.1.1 Electronic Structure

At the equilibrium geometry, the S_1_ state has nπ* character and S_2_ is a ππ* state (see summary in Figure 5 in the Supporting Information).^37^ Figure 1 shows an LIIC that traces the ground- and excited-state energies from the S_0_ equilibrium geometry to the optimized S_2_/S_1_ MECI and then onward to the S_1_/S_0_ MECI. The three key molecular geometries are shown at the bottom of Figure 1, evidencing the ring-opening nature of this reaction coordinate. At the equilibrium geometry, the C–O bond is 1.38 Å, which stretches to 2.30 Å at the S_2_/S_1_ MECI. The changes going to the S_1_/S_0_ MECI are comparatively small and involve further extension of the C–O bond to 3.06 Å.

**Figure 1:**
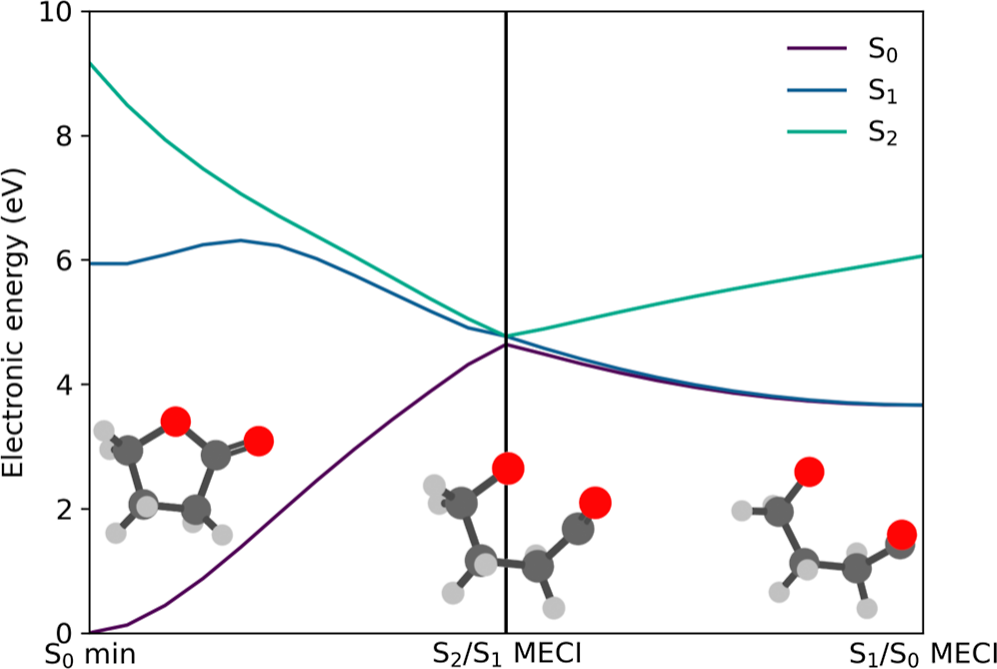
Ground- and excited-state potential energy curves for states S_0_ (purple), S_1_ (blue), S_2_ (green) along the LIIC for γ-butyrolactone, calculated at the SA(3)-CASSCF­(10,8)/cc-pVDZ level of theory. The LIIC starts at the ground-state geometry S_0_ minimum (Left). It then proceeds to the α-C–O breaking S_2_/S_1_ MECI (Center). Finally, the LIIC terminates at the α-C–O ring-open S_1_/S_0_ MECI (Right). A vertical solid black line separates the left and right segments of the LIIC and the three molecular structures S_0_ min, S_2_/S_1_ MECI, S_1_/S_0_ MECI are shown as insets.

The energies in Figure 1 are calculated at the SA(3)-CASSCF­(10,8)/cc-pVDZ level of theory. Upon photoexcitation to the S_2_ state, the LIIC indicates a steep gradient toward the ring-opened structure via the S_2_/S_1_ MECI, resulting in a rupture of the C–O bond. The dissociative character arises from out-of-plane motion coupled to the C–O bond extension, destabilizing the π orbital while stabilizing the π* orbital. The same dissociative behavior is observed for XMS(3)-CASPT2­(10,8), as shown in Figure S6 in the Supporting Information, albeit with a less steep gradient (as a result of SA-CASSCF recovering static correlation but not dynamic correlation). At the SA(3)-CASSCF­(10,8) level, the S_2_/S_1_ MECI energy is 4.40 eV below the vertical S_2_ energy at the equilibrium geometry. However, the rapid stabilization of S_2_ and the commensurate destabilization of S_1_ along the LIIC means that S_1_ and S_2_ quickly acquire a mixed nπ* and ππ* character when they approach the S_2_/S_1_ MECI. Beyond the S_2_/S_1_ MECI, the S_2_ state is initially dominated by the S_0_ equilibrium configuration but acquires significant σ* character, while the S_0_ and S_1_ states now carry the mixed *n*π* and ππ* character.

#### 4.1.2 Nonadiabatic Simulations

This section presents the simulations of γ-butyrolactone used to calculate the time-dependent UED signals in Section 4.3. Figure 2a shows the adiabatic populations for the three lowest-lying singlet states, S_0_–S_2_. At *t* = 0 fs, the trajectories are initiated on the S_2_ state, with rapid population transfer to the S_1_ state consistent with the LIIC reaction coordinate in Figure 1 and the mechanism proposed in previous literature.^37^ A rise in ground-state population follows soon after the increase in S_1_ population, commensurate with the close proximity of the S_1_/S_0_ and S_2_/S_1_ MECIs. As the S_1_ and S_0_ states are close in energy after the S_2_/S_1_ MECI (Figure 1), the accuracy of FSSH is likely reduced after the S_2_/S_1_ MECI is reached.

**Figure 2:**
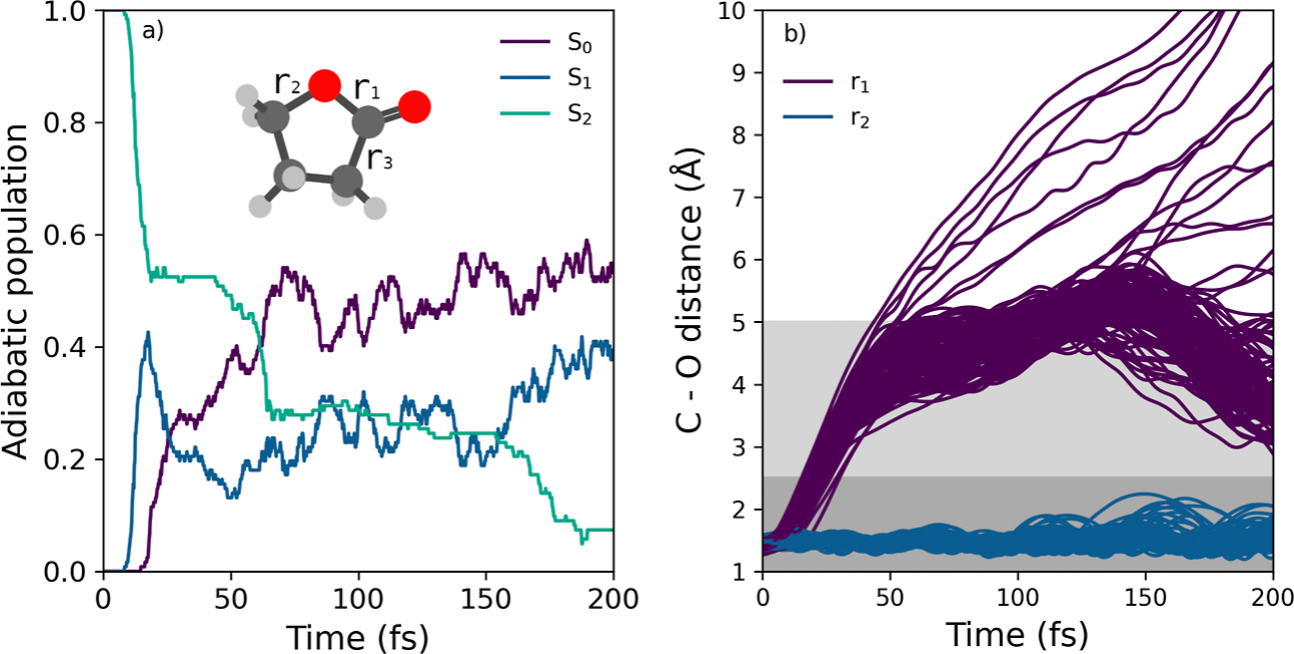
TSH Simulation for γ-butyrolactone for times *t* ∈ [0, 200] fs. (a) Time-dependent classical adiabatic populations for three singlet electronic states, S_2_ (green), S_1_ (blue) and S_0_ (purple). The inset shows the three key bonds involved in the dynamics at the equilibrium geometry of the molecule (*r*_1_, *r*_2_, and *r*_3_). (b) Time-evolution of the *r*_1_ and *r*_2_ C–O bond lengths for all 122 trajectories. Three ranges of C–O distances are highlighted by different background colors. Dark gray background (bottom) where bonds are considered bound, light gray (middle) indicates a single C–O bond broken, and white background corresponds dissociation of a CO fragment.

The two C–O bonds within the five-membered ring are labeled *r*_1_ and *r*_2_, and the α-C–C bond is labeled *r*_3_ (see inset in Figure 2a). Bonds *r*_1_ and *r*_2_ are plotted for all trajectories as a function of time in Figure 2b. The *r*_1_ distances (purple) exhibit immediate elongation corresponding to the ring-opening reaction, which largely takes place on the S_2_ state as anticipated from the LIIC in Figure 1. After around 50 fs, two distinct subsets appear within the *r*_1_ traces, with some trajectories exhibiting large increases in the *r*_1_ bond distance to values beyond 5 Å. Examination of the adjacent C–C bond (*r*_3_) for this subset confirms that, for these trajectories, the *r*_3_ bond also breaks. This second C–C bond breaking occurs on the S_0_ ground state, sequentially with the initial C–O (*r*_1_) bond break. Three regions are thus indicated in Figure 2b: (i) the dark gray background ( <2.5⁡ Å) contains the intact *r*_2_ bond lengths, (ii) the light gray background (2.5–5.0 Å) contains the trajectories where the *r*_1_ bond breaks to yield a ring-open molecule, and (iii) the white background ( <5.0⁡ Å) contains the dissociative trajectories where two bonds have been broken to yield CO and CH_2_CH_2_CH_2_O fragments. Finally, all *r*_2_ bonds (blue) remain within the dark gray region of Figure 2b. This indicates that these bonds remain intact throughout the simulation, although oscillations increase in amplitude at later times (>150fs) . A full summary of the quantum yields for each process is given in Table S3.

### 4.2 Static Scattering

#### 4.2.1 Scattering at Equilibrium Geometry

We begin the investigation of the scattering signals by examining the scattering from the electronic ground state of γ-butyrolactone at the equilibrium geometry. Figure 3 shows the static scattering signal calculated using the IAM and AIS using CCSD and SA(3)-CASSCF­(10,8) wave functions, shown as Δ*I*(*s*) = *I*_method_(*s*) – *I*_CCSD_(*s*). The most correlated method, CCSD, is taken as the reference given the sensitivity of scattering to electron correlation.^61^ Ideally, a larger basis than cc-pVDZ should be used to recover more electron correlation. However, we use cc-pVDZ to maintain consistency with the level of theory used to calculate the scattering from the TSH simulations. Due to the high-quality description of the bonding environment provided by CCSD at equilibrium geometries, where the ground state is dominated by a single determinant, we expect CCSD to yield reference-quality scattering signals.^61^ Looking at the Δ*I*(*s*) scattering curves in Figure 3, we observe significant differences between the IAM and the AIS. The largest deviation from CCSD clearly appears for the IAM, with the maximum error for SA(3)-CASSCF­(10,8) two-thirds smaller. The effects of the active space size are shown in Section Active spaces in the Supporting Information. Although SA(3)-CASSCF­(10,8) only includes a small amount of dynamic correlation, it does account for chemical bonding and polarization within the molecule, which the IAM cannot. The largest improvements for CASSCF, as compared to IAM, are observed in the range $2\;{Å}^{-1}<s<5\;{Å}^{-1}$ . This is commensurate with what has been observed previously in X-ray scattering and maps onto real-space length scales where correlation effects and chemical bonding manifest. The discrepancies persist up to $s=8\;{Å}^{-1}$ , but are most apparent at smaller momentum transfer values.

**Figure 3:**
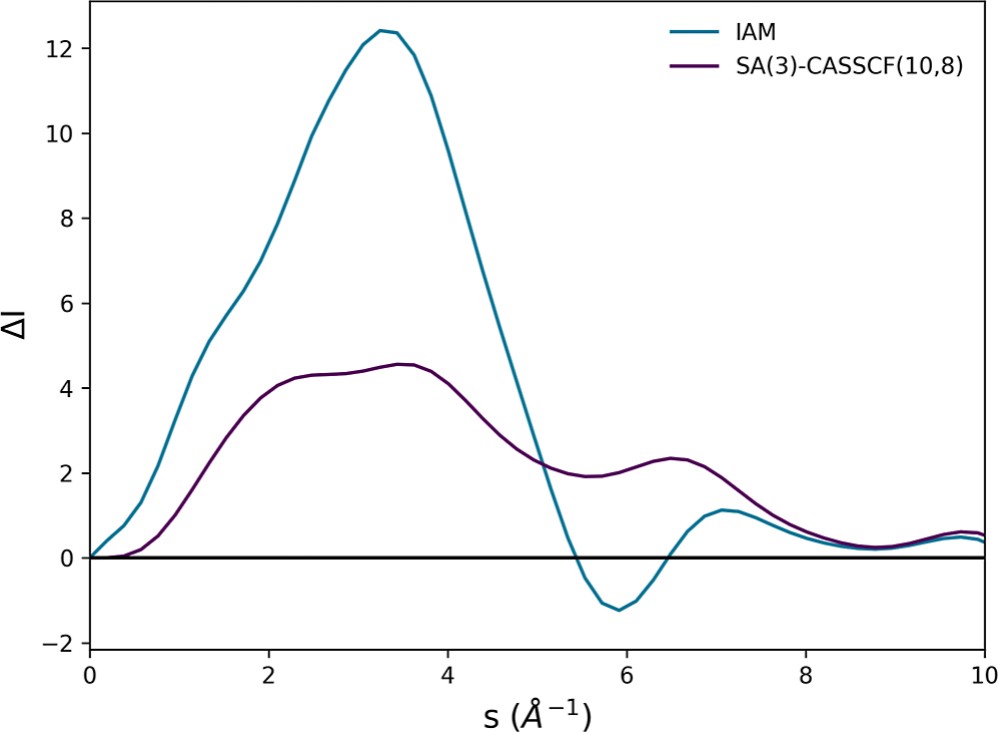
Differences in the scattering probability, Δ*I*, for γ-butyrolactone at the S_0_ equilibrium geometry shown in Figures 1 and 2. The differences are shown relative to the AIS obtained from CCSD/cc-pVDZ for both SA-CASSCF­(10,8)/cc-pVDZ and from the IAM.

#### 4.2.2 Scattering along LIIC

Next, we calculate the total isotropic scattering signals from the ground and excited electronic states S_0_–S_2_ along the LIIC described in Section 4.1 using SA(3)-CASSCF­(10,8)/cc-pVDZ. Figure 4 shows the percent difference scattering for the S_2_ and S_1_ states calculated according to eq 5, with the scattering from the S_0_ state taken as the reference. Note that the region $s<0.75\;{Å}^{-1}$ is not shown to avoid numerical artifacts arising from the small denominator. Figure 4 reveals how much the scattering signals of the two excited states differ from the ground state at each point along the LIIC. At the equilibrium geometry, the S_2_ (ππ*) state in panel a displays a positive difference of ∼5% at small momentum transfer $s<1.5\;{Å}^{-1}$ , while the S_1_ (*n*π*) state in panel b has negative values of similar magnitude. The differences between the scattering from the three electronic states are greatest at the equilibrium geometry and decrease along the LIIC. We attribute this to the S_2_ state having almost pure ππ* character at equilibrium, resulting in a strong redistribution of the electrons compared to the ground state (see Figure S5) and a sizable percent difference. The largest differences appear for $s<5\;{Å}^{-1}$ , consistent with what was observed in Figure 3. It is worth noting that excited state scattering can differ even more from the ground state for e.g. Rydberg^6,31,35,49,95^ and charge-transfer states.^33^

**Figure 4:**
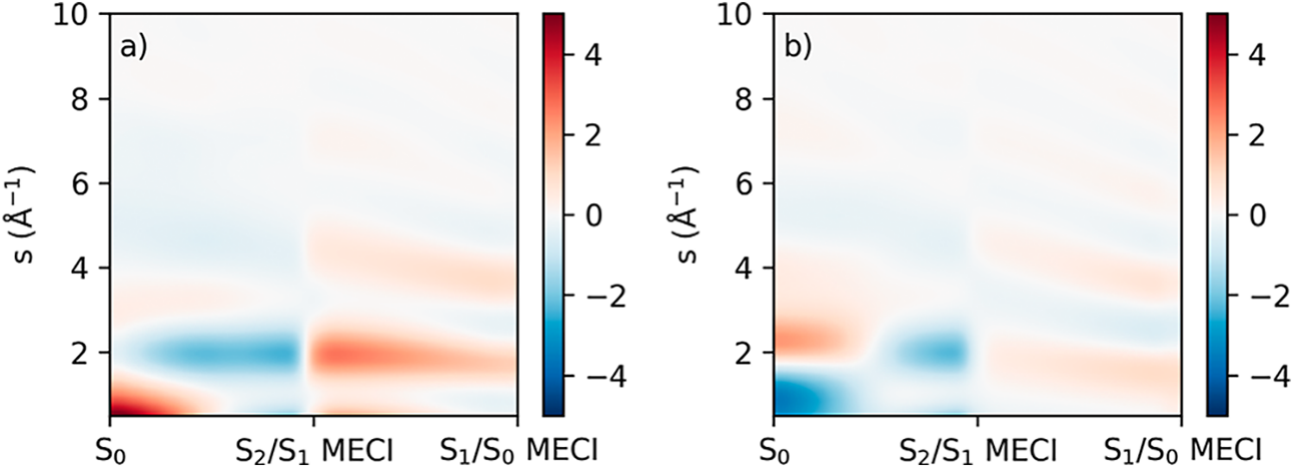
Total isotropic percent difference scattering signals as functions of momentum transfer *s* of γ-butyrolactone along the LIIC in Figure 1. The signals are defined relative to the electronic ground state for (a) the signal of the S_2_ state. (b) The signal of the S_1_ state.

As we move toward the S_2_/S_1_ MECI, the strong feature at small *s* in Figure 4a diminishes and becomes weakly negative due to the mixing of the *n*π* and ππ* characters of the S_2_ and S_1_ states discussed in Section 4.2.2. Since the LIIC path consists of two segments, one from the equilibrium to the S_2_/S_1_ MECI and one between the S_2_/S_1_ and S_1_/S_0_ MECIs, there is a distinct discontinuity at the S_2_/S_1_ MECI in both panels.

A region of strong negative percent difference is centered at around $s\approx 2\;{Å}^{-1}$ in the first segment of Figure 4a and increases in intensity until it abruptly changes sign at the S_2_/S_1_ MECI. This is due to the crossing of the diabatic states at the CI, where the adiabatic S_2_ state acquires significant ground-state character as reflected by a large (0.58) CI coefficient for the ground-state determinant for geometries beyond the S_2_/S_1_ MECI. Moreover, there is a region in the immediate vicinity of the S_2_/S_1_ MECI where the percent differences are small due to the near-degeneracy of the adiabatic states. In the second segment, the positive percent difference diminishes toward the S_1_/S_0_ MECI where the S_0_ state acquires more ππ* character, while the S_2_ remains strongly mixed with the initial ground-state character. Interestingly, in the first half of the LIIC in Figure 4b, there is a sign change in the percent difference about the midpoint of that segment. This is likely caused by the S_1_ state’s diabatic character becoming increasingly mixed with that of the S_2_ state, i.e. a combination of *n*π* and ππ*.

The second segment in both panels a and b in Figure 4 exhibits less structure due to the smaller effective changes in molecular geometry and the associated small changes in the electronic character as the S_2_ state gains *n*σ* character (see Section 4.1). Equally, the mixed *n*π* and ππ* character of S_1_ past the S_2_/S_1_ MECI results in only minor changes to the features in Figure 4b with an overall positive percent difference.

### 4.3 Time-Resolved Scattering

We now proceed to investigate the scattering signals for time-resolved UED, calculated from the full simulations presented in Section 4.1. We compare AIS and IAM signals as percent differences obtained using eq 6, with the former based on SA(3)-CASSCF­(10,8)/cc-pVDZ. To ensure like-for-like comparison that is unaffected by experimental errors, the comparison is made between theoretical signals where the UED observable is calculated at different levels of approximation/theory.

The total AIS and IAM signals are shown in Figure 5a,b. At first sight, the two signals appear very similar, reflecting that the IAM captures the main features of the time-resolved UED signal rather well. Closer inspection reveals a distinct positive (red) feature in the lower left corner of panel a ( $s<1.5\;{Å}^{-1}$ , *t* < 15 fs), which is absent from the IAM (panel b). This is due to the instantaneous population of the S_2_ electronic state at *t* = 0 fs in the sudden approximation, and is consistent with the feature observed in Figure 4a. It is a weaker version of the scattering signal observed previously for low-lying Rydberg states,^6,31,35^ commensurate with the smaller change of a ππ* transition. The feature vanishes after approximately 15 fs due to rapid early times dynamics.

**Figure 5:**
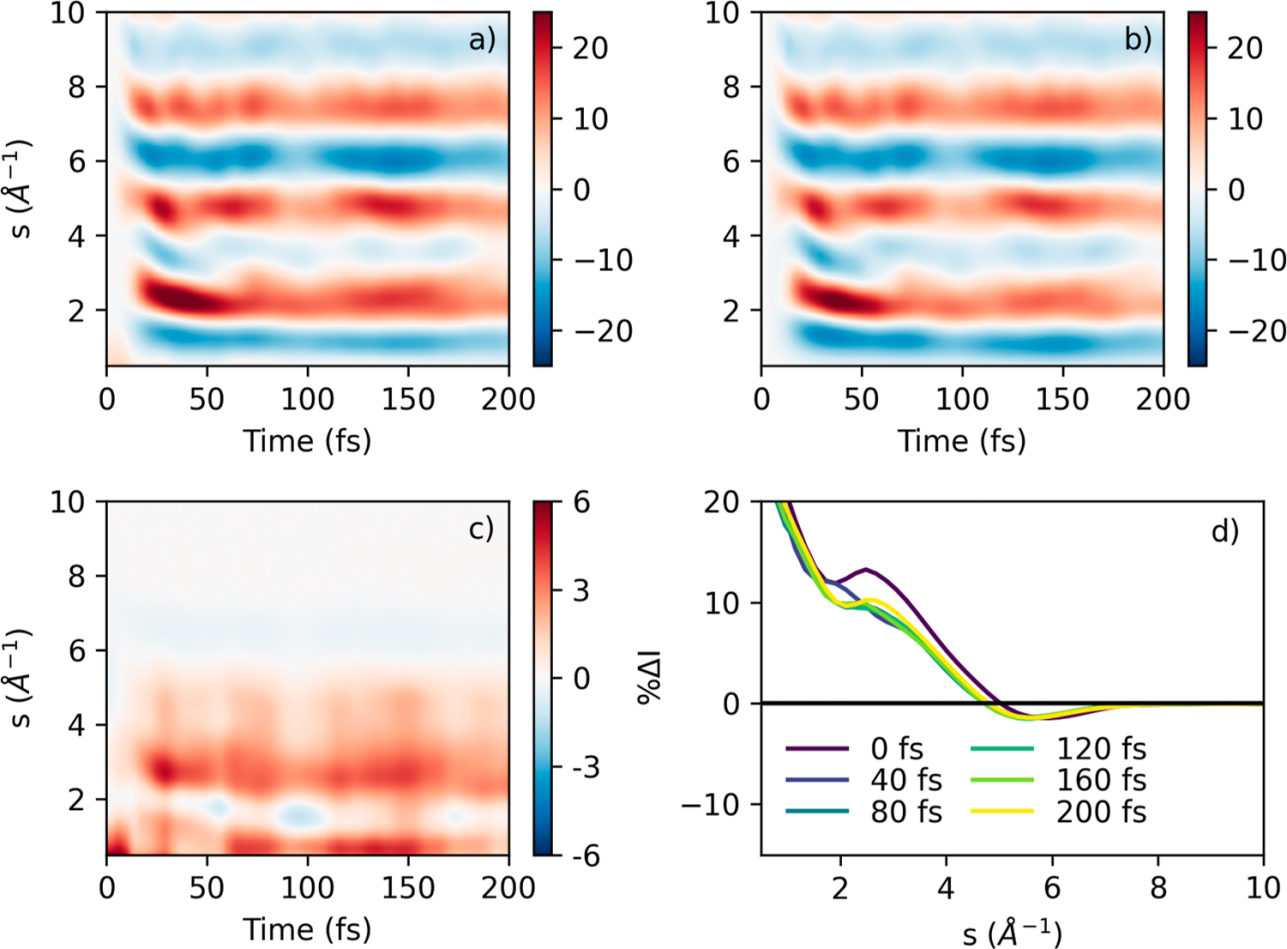
Total isotropic UED signal, %Δ*I*_method_(*s*, *t*), as a function of momentum transfer *s* and time *t*, calculated using eq 6 and all *N*_trj_ = 122 trajectories. (a) The AIS signal accounting for the active state of each trajectory at each time, calculated at the SA(3)-CASSCF­(10,8)/cc-pVDZ level of theory. (b) The IAM scattering, calculated using the same molecular geometries as in a. (c) The difference between the signals in panels a and b, as %ΔΔ*I*(*s*, *t*) = %Δ*I*_AIS_(*s*, *t*) – %Δ*I*_IAM_(*s*, *t*). (d) The percent difference between the IAM and the AIS signals at intervals of Δ*t* = 40 fs, with the AIS at each time taken as the reference.

More detailed discrepancies between AIS and the IAM can be discerned if one considers the difference %ΔΔ*I*(*s*, *t*) = %Δ*I*_AIS_(*s*, *t*) – %Δ*I*_IAM_(*s*, *t*) in Figure 5c, showing persistent differences of up to 6% for $s<6\;{Å}^{-1}$ at all times. As before, these differences can be attributed to the redistribution of electrons due to chemical bonding and associated adjustments in electron correlation. Comparing the %ΔΔ*I*(*s*, *t*) difference in panel c with the AIS and IAM signals in panels a and b reveals that, although the overall patterns of the IAM and AIS are similar, their intensities are quite different. Considering that the overall magnitudes of the IAM/AIS percent difference signals are on the order of ±30%, the 6% difference is significant. The greatest differences appear in the range 2 < *s* < 4 Å^–1^, just like in the static results in Figure 4. The predominantly small-*s* differences echo the conclusion in ref 96 that the scattering signal at larger momentum transfer provides a clearer representation of the molecular geometry less affected by electronic effects.

Finally, the straight IAM vs AIS percent difference, with AIS taken as the reference, is shown in Figure 5d, with lineouts taken at 40 fs intervals throughout the simulation. Again, the largest differences are observed at small values of *s*. Comparing Figure 5d with the LIIC curves in Figure S15, we find that these are quite similar and that sizable discrepancies between IAM and AIS persist in the full simulation, especially for < 6 Å^–1^. The *t* = 0 line differs the most, reflecting that the greatest changes in electron density occur during the initial dissociation of the intraring C–O bond (*r*_1_). After the *r*_1_ bond is broken, differences in the molecular structure yield smaller changes in the valence electron density, leading to more consistent differences between the IAM and the AIS signals at later times (*t* > 30 fs).

#### 4.3.1 Calculating Time-Constants

As a demonstration of the error incurred from the IAM, we isolate two lineouts from the AIS and IAM scattering signals in Figure 5 at *s* = (1.3, 2.3) ${Å}^{-1}$ . These two lineouts correspond to strong early time features in the signal and represent the type of distinct peaks that would often be used to determine time-zero and initial time-constants for the dynamics. For the *s* = 2.3 Å^–1^ curve, the differences between AIS and the IAM are very small for *t* ≤ 12 fs. However, for *t* > 12 fs, the two signals diverge by ∼ 6% as noted earlier. In contrast, for the *s* = 1.3 Å^–1^ curve, the difference between the IAM and AIS fit is significant already from the beginning, commensurate with the sudden excitation to the S_2_ state (see also Figures 4 and 5). For *t* ≤ 16 fs, the difference is on the order of 2–3%, while for *t* > 16 fs the difference increases due to a combination of a redistribution of valence electrons during the ring-opening and the effect of the electronic states on the scattering intensity.

The calculated data for the IAM and AIS in Figure 6 is subsequently fit to an error function

7 $$I_{fitted}\left(t\right)=A\left(1+\mathrm{erf}\left(\frac{t-t_{0}}{\tau }\right)\right)$$

with three parameters: scaling factor *A*, the center *t*_0_, and lifetime τ. The fitted parameters are given in Table 1 and the corresponding error functions are shown as solid lines in Figure 6. The time-constants are commensurate with the rapid dynamics, a direct result of the ultrafast ring-opening in γ-butyrolactone. The *s* = 1.3 Å^–1^ curve yields a lifetime of 8.8 fs for the IAM and 6.4 fs for the AIS. The *s* = 2.3 Å^–1^ curve has an even faster rise, with IAM and AIS lifetimes of 6.9 and 6.2 fs, respectively. Although the absolute differences between the IAM and AIS are comparatively small, the percent changes are significant (28.8% for *s* = 1.3 Å^–1^ and 11.3% for *s* = 2.3 Å^–1^). The mean absolute percent error (MAPE) of all fitted parameters

$$MAPE=\frac{100}{N}\overset{N}{\underset{i=1}{\sum }}\frac{\left|\gamma_{i}^{AIS}-\gamma_{i}^{IAM}\right|}{\gamma_{i}^{AIS}}$$ 8

is taken. Where γ_*i*_^IAM^ and γ_*i*_^AIS^ are the *i*th IAM and AIS parameters, respectively. The calculated MAPE yields a value of 12% for all parameters. Within this analysis, we note that the error for the centers of the fits is significantly lower than that of the lifetimes. This is a consequence of the fact that the difference between the IAM and AIS mostly manifests in intensity of the signal rather than in the positions of the scattering features. A temporal convolution with a 130 fs fwhm Gaussian is shown in Figure S20, demonstrating that the differences between IAM and AIS persist. This result demonstrates the importance of the manner in which observables are calculated when making predictions based on simulations, or when comparing simulations and experiments.

**Figure 6:**
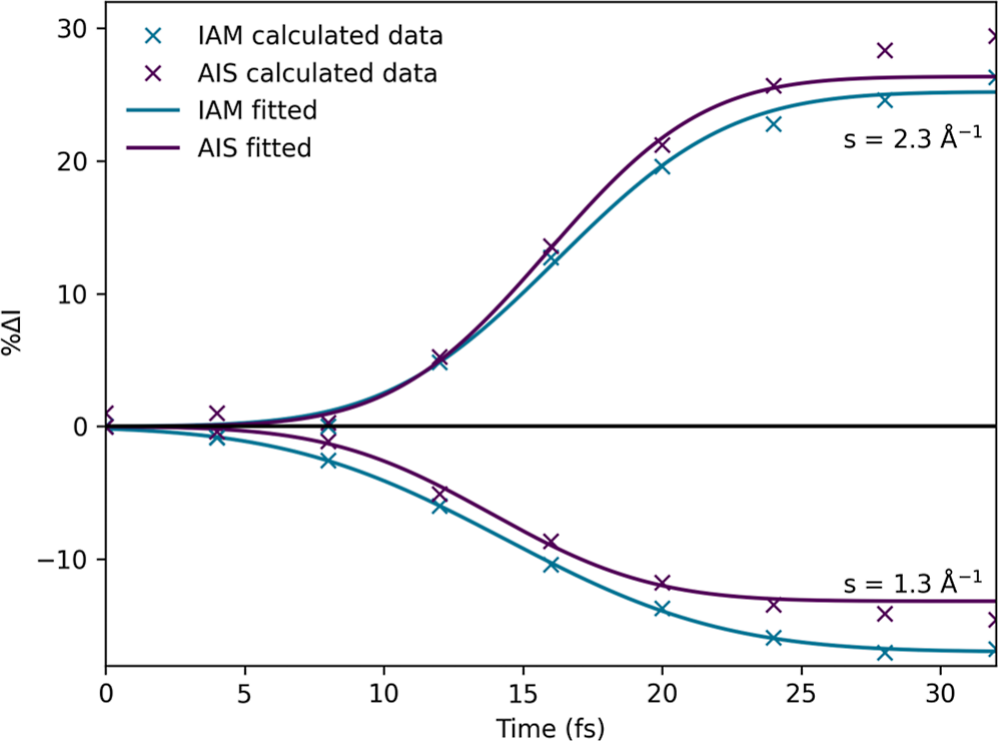
Lineouts of the IAM and AIS %Δ*I*(*s*, *t*) signals in Figure 5a,b at $s=1.3\;{Å}^{-1}$ and $s=2.3\;{Å}^{-1}$ as a function of time, with data shown as purple crosses for the AIS and green crosses for the IAM. The fit to an error function, eq 7, is shown by solid lines in the corresponding color.

**Table 1:** A Summary of the Parameters in eq 7 Fitted to the Lineouts of the UED Signals Shown in Figure 6

	$s=1.3\;{Å}^{-1}$			$s=2.3\;{Å}^{-1}$		
method	A	center (*t*_0_)	lifetime (τ)	A	center (*t*_0_)	lifetime (τ)
IAM	–8.5	14.4	8.8	12.6	16.2	6.9
AIS	–6.6	13.9	6.4	13.2	15.9	6.2
difference (%)	28.8	3.6	21.8	4.5	1.9	11.3

#### 4.3.2 Decomposing the Signal for Reaction Channels

The presence of two distinct reaction channels in the dynamics, one leading to ring-opening and the other to CO dissociation (see Section 4.1), provides a further opportunity to examine the scattering signals. We split the scattering signals in Figure 5 into two sets corresponding to the two reaction channels. The pump-off reference signal, *I*_method_(*s*, *t* < 0), is calculated using the full set of 122 trajectories, while %Δ*I*_method_(*s*, *t*) is renormalized for each set according to the number of trajectories in that set. This underpins the very small percent differences in the AIM signal at *t* = 0.

Figure 7 shows the IAM and AIS percent difference signals at *t* = 0 and *t* = 200 fs, i.e. immediately following the sudden excitation and at the end of the simulation (the full signals for each channel are shown in the Supporting Information Section Clusters). At *t* = 0 fs (Figure 7a), the IAM signals are essentially zero. In contrast, the AIS signals have percent differences of ∼ 5.5% due to being situated on the S_2_ state, as seen in Figure 5a previously. An alternative separation of the signal into nuclear and electronic contributions, outlined in ref 31, is provided in the Supporting Information. At *t* = 200 fs, the differences between the two reaction channels are more striking, reflecting the structural differences between the two channels. The differences between IAM and AIS in the region 2 Å^–1^ < *s* < 5 Å^–1^ are nearly 3%, close to the actual magnitude of the signal itself.

**Figure 7:**
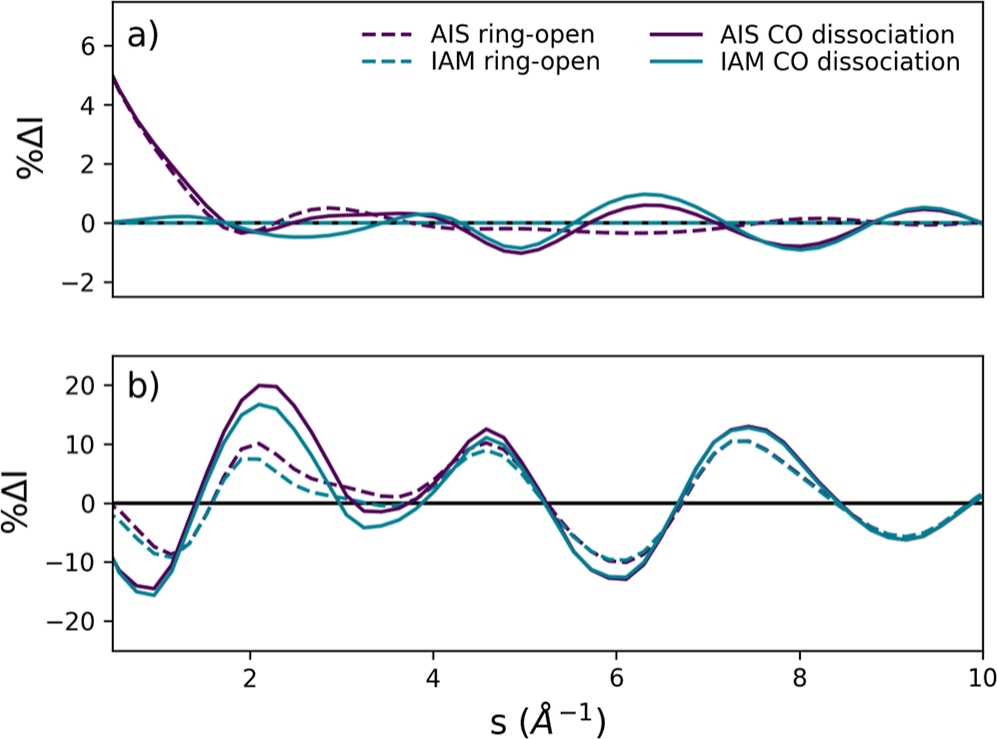
Total isotropic scattering for the two sets of trajectories, corresponding to ring-opening (dashed lines) and CO dissociation (solid lines). Scattering shown as lineouts at times *t* = 0 (panel a) and *t* = 200 fs (panel b) as a function of momentum transfer *s*. The percent differences are calculated using eq 6 (see text for further details). The results are shown for ring-open and CO dissociation calculated with AIS (purple) and the IAM (blue).

## 5 Conclusions

This work highlights the importance of accurate calculations of experimental observables for time-resolved UED. Crucially, we find that the discrepancies between the independent atom model (IAM) and *ab initio* scattering (AIS) survive the averaging over molecular structures during the dynamics.

The IAM reproduces the overall shape of the scattering signal well, with the obvious exception of excited-state signatures, at a very low computational cost. Using AIS modifies the intensities of the peaks and troughs in the percent difference scattering by up to ∼ 6%, a significant fraction of the overall ±30% signal. The discrepancies reflect electronic effects such as chemical bonding, electronic states, and electron correlation that are absent in the IAM. Moreover, these differences are sufficient to affect lifetimes and other parameters determined from the scattering and suggest that usage of the IAM could yield systematic errors when inverting scattering signals.^97–101^ The differences observed in signal intensity are likely to be measurable,^9,102^ but the differences observed in lifetimes are unlikely to be so.

Our static benchmarks, which extend beyond the Franck–Condon region to cover the whole reaction coordinate, reveal that changes of the predicted scattering signals due to the choice of the electronic structure method are significantly smaller than the differences between the IAM and AIS. For instance, as long as the active space is reasonable, different active spaces give similar results, with the differences mainly contained at small values of *s*. This result can be anticipated. Once the dominant determinants are included, the wave function accounts for most of the static correlation and the amount of dynamic correlation included in CASSCF is small. We note that our CCSD calculations illustrate that the inclusion of dynamic correlation is needed to achieve highly accurate UED signals, especially at small and intermediate values of *s*.

The differences between the ground and excited electronic states manifest at small values of *s* and can be significant. In the present study, the scattering signals of the excited *n*π* and ππ* states of γ-butyrolactone differ by ±5% from the ground state, and one may anticipate even greater differences for Rydberg and charge transfer states.

In terms of best practice, there is a strong argument for using AIS to predict UED signals. However, such computations can be expensive, AIS calculations on γ-butyrolactone take 10 min per point on 4 cores with Intel­(R) Xeon­(R) Gold 6326 CPU @ 2.9 GHz, in contrast to seconds for the full set of IAM calculations. Our results suggest that the shortcomings of the IAM can be assessed by examining static benchmarks along representative LIIC’s. This also provides an opportunity to evaluate different choices of electronic structure methods for the AIS. Before using AIS to predict the UED signal from a full dynamics simulation, one may also consider calculating AIS along representative trajectories,^103,104^ possibly selected using clustering methods, as experimental scattering signals can often be reproduced with select representative trajectories.^105,106^ Here, we also note that when comparing to an experiment, careful attention must be paid to ensure that the simulation of the excited state dynamics is accurate as possible.^79–84^

Interesting future work along the lines of this paper would include detailed comparison between experimental data, both static and time-resolved, against various levels of theory. This would require careful calibration of the experimental data to remove systematic biases and distortions.^107^ More broadly, benchmarks for other observables could be investigated. Most closely related to the current study is ultrafast X-ray scattering (UXS), where there are already extensive static benchmarks,^42,43,45,51,61^ but also other observables such as time-resolved photoelectron spectroscopy or transient (X-ray) absorption would be of interest.^108–110^ One consideration of such studies would be the different time-resolution currently achievable by these different methods.

We conclude with the strongest argument for AIS calculations. Scattering signals beyond the IAM are necessary when considering scattering experiments that probe electronic effects alongside the structural dynamics in photoexcited molecules. Improvements in experimental capabilities and facilities increasingly allow such details to be extracted from experimental observations, including the signatures of the electronic states involved in the dynamics,^6,31,34,35^ changes in electronic state populations,^32^ charge transfer,^33^ and kinetics.^111^ These experimental advances simply must be matched by more sophisticated and accurate methods for predicting the observables. The continued improvements in UED and UXS thus make high-accuracy simulations of vital importance for the interpretation of experimental data.

## Supplementary Material



## Notes

Details on the active space used; effects of the *t* = 0 fs reference signal on the overall signal; benchmarking of electronic structure methods; example trajectories; quantum yields of the processes; effects of the active space on the UED signals; the role of basis sets on the signal; decomposition of the signal into elastic and inelastic components; decomposition of the signal for all reaction pathways; the effects of relativistic corrections on the signal; decomposition of the signal into the electronic and nuclear components; lifetimes obtained after convolutions with the probe pulse (PDF)

## †

Instituto de Ciencia Molecular, Universitat de València, Apartado22085, ES-46071 Valencia, Spain

## References

[1] Centurion M., Wolf T. J., Yang J. (2022). Ultrafast Imaging of Molecules with
Electron Diffraction. Annu. Rev. Phys. Chem..

[2] Filippetto D., Musumeci P., Li R., Siwick B., Otto M., Centurion M., Nunes J. (2022). Ultrafast electron diffraction: Visualizing
dynamic states of matter. Rev. Mod. Phys..

[3] Ischenko A. A., Weber P. M., Miller R. J. D. (2017). Capturing Chemistry
in Action with
Electrons: Realization of Atomically Resolved Reaction Dynamics. Chem. Rev..

[4] Ishikawa T., Hayes S. A., Keskin S., Corthey G., Hada M., Pichugin K., Marx A., Hirscht J., Shionuma K., Onda K., Okimoto Y., Koshihara S.-y., Yamamoto T., Cui H., Nomura M., Oshima Y., Abdel-Jawad M., Kato R., Miller R. J. D. (2015). Direct
observation
of collective modes coupled to molecular orbital-driven charge transfer. Science.

[5] Minitti M. P., Budarz J. M., Kirrander A., Robinson J. S., Ratner D., Lane T. J., Zhu D., Glownia J. M., Kozina M., Lemke H. T., Sikorski M., Feng Y., Nelson S., Saita K., Stankus B., Northey T., Hastings J. B., Weber P. M. (2015). Imaging Molecular
Motion: Femtosecond X-Ray Scattering
of an Electrocyclic Chemical Reaction. Phys.
Rev. Lett..

[6] Stankus B., Yong H., Zotev N., Ruddock J. M., Bellshaw D., Lane T. J., Liang M., Boutet S., Carbajo S., Robinson J. S., Du W., Goff N., Chang Y., Koglin J. E., Minitti M. P., Kirrander A., Weber P. M. (2019). Ultrafast X-ray scattering reveals
vibrational coherence
following Rydberg excitation. Nat. Chem..

[7] Wolf T. J., Sanchez D. M., Yang J., Parrish R. M., Nunes J. P., Centurion M., Coffee R., Cryan J. P., Gühr M., Hegazy K., Kirrander A., Li R. K., Ruddock J., Shen X., Vecchione T., Weathersby S. P., Weber P. M., Wilkin K., Yong H., Zheng Q., Wang X. J., Minitti M. P., Martínez T. J. (2019). The photochemical
ring-opening of 1,3-cyclohexadiene imaged by ultrafast electron diffraction. Nat. Chem..

[8] Stefanou M., Saita K., Shalashilin D. V., Kirrander A. (2017). Comparison
of Ultrafast Electron and X-Ray Diffraction – A Computational
Study. Chem. Phys. Lett..

[9] Ma L., Yong H., Geiser J. D., Moreno Carrascosa A., Goff N., Weber P. M. (2020). Ultrafast x-ray and electron scattering
of free molecules: A comparative evaluation. Struct. Dyn..

[10] Simmermacher, M. ; Weber, P. M. ; Kirrander, A. Structural Dynamics with X-ray and Electron Scattering; Royal Society of Chemistry, 2023; pp 85–125.

[11] Debye P. (1930). X-ray interference
patterns and atomic dimensions. Phys. Zeits..

[12] Heisenberg W. (1931). Über
die inkohärente Streeung von Röntgenstrahlen. Phys. Zeits..

[13] Morse (1932). Unelastische Streuung von Kathodenstrahlen. Phys. Zeits..

[14] Bewilogua (1933). Über die Streuung von Röntgen-und
Kathodenstrahlen an freien Molekülen. Phys. Zeits..

[15] Martinez, T. J. ; Wolf, T. ; Slavíček; Worth, G. ; Barbatti, M. ; Curchod, B. ; Bonella, S. Prediction Challenge: Cyclobutanone Photochemistry. https://pubs.aip.org/collection/16531/Prediction-Challenge-Cyclobutanone-Photochemistry (accessed Jan 2026). 10.1063/5.0344957 42504898

[16] Vindel-Zandbergen P., González-Vázquez J. (2024). Non-adiabatic dynamics of photoexcited
cyclobutanone: Predicting structural measurements from trajectory
surface hopping with XMS-CASPT2 simulations. J. Chem. Phys..

[17] Miller E. R., Hoehn S. J., Kumar A., Jiang D., Parker S. M. (2024). Ultrafast
photochemistry and electron diffraction for cyclobutanone in the S2
state: Surface hopping with time-dependent density functional theory. J. Chem. Phys..

[18] Martín
Santa Daría A., Hernández-Rodríguez J., Ibele L. M., Gómez S. (2024). Photofragmentation of cyclobutanone
at 200 nm: TDDFT vs CASSCF electron diffraction. J. Chem. Phys..

[19] Miao X., Diemer K., Mitrić R. (2024). A CASSCF/MRCI
trajectory surface
hopping simulation of the photochemical dynamics and the gas phase
ultrafast electron diffraction patterns of cyclobutanone. J. Chem. Phys..

[20] Suchan J., Liang F., Durden A. S., Levine B. G. (2024). Prediction challenge:
First principles simulation of the ultrafast electron diffraction
spectrum of cyclobutanone. J. Chem. Phys..

[21] Janoš J., Figueira Nunes J. P., Hollas D., Slavíček P., Curchod B. F. (2024). Predicting the photodynamics of cyclobutanone triggered
by a laser pulse at 200 nm and its MeV-UED signals—A trajectory
surface hopping and XMS-CASPT2 perspective. J. Chem. Phys..

[22] Eng J., Rankine C. D., Penfold T. J. (2024). The photochemistry
of Rydberg-excited
cyclobutanone: Photoinduced processes and ground state dynamics. J. Chem. Phys..

[23] Mukherjee S., Mattos R. S., Toldo J. M., Lischka H., Barbatti M. (2024). Prediction
Challenge: Simulating Rydberg photoexcited cyclobutanone with surface
hopping dynamics based on different electronic structure methods. J. Chem. Phys..

[24] Makhov D. V., Hutton L., Kirrander A., Shalashilin D. V. (2024). Ultrafast
electron diffraction of photoexcited gas-phase cyclobutanone predicted
by ab initio multiple cloning simulations. J.
Chem. Phys..

[25] Jaiswal V. K., Montorsi F., Aleotti F., Segatta F., Keefer D., Mukamel S., Nenov A., Conti I., Garavelli M. (2024). Ultrafast
photochemistry and electron-diffraction spectra in n → (3s)
Rydberg excited cyclobutanone resolved at the multireference perturbative
level. J. Chem. Phys..

[26] Bennett O., Freibert A., Spinlove K. E., Worth G. A. (2024). Prediction through
quantum dynamics simulations: Photo-excited cyclobutanone. J. Chem. Phys..

[27] Lawrence J. E., Ansari I. M., Mannouch J. R., Manae M. A., Asnaashari K., Kelly A., Richardson J. O. (2024). A MASH
simulation of the photoexcited
dynamics of cyclobutanone. J. Chem. Phys..

[28] Hutton L., Moreno Carrascosa A., Prentice A. W., Simmermacher M., Runeson J. E., Paterson M. J., Kirrander A. (2024). Using a multistate
mapping approach to surface hopping to predict the ultrafast electron
diffraction signal of gas-phase cyclobutanone. J. Chem. Phys..

[29] Peng J., Liu H., Lan Z. (2024). The photodissociation
dynamics and ultrafast electron
diffraction image of cyclobutanone from the surface hopping dynamics
simulation. J. Chem. Phys..

[30] Hait D., Lahana D., Fajen O. J., Paz A. S., Unzueta P. A., Rana B., Lu L., Wang Y., Kjønstad E. F., Koch H., Martínez T. J. (2024). Prediction
of photodynamics of 200
nm excited cyclobutanone with linear response electronic structure
and ab initio multiple spawning. J. Chem. Phys..

[31] Yong H., Zotev N., Ruddock J. M., Stankus B., Simmermacher M., Carrascosa A. M., Du W., Goff N., Chang Y., Bellshaw D., Liang M., Carbajo S., Koglin J. E., Robinson J. S., Boutet S., Minitti M. P., Kirrander A., Weber P. M. (2020). Observation of the molecular response to light upon
photoexcitation. Nat. Commun..

[32] Yang J., Zhu X., Nunes J. P. F., Yu J. K., Parrish R. M., Wolf T. J. A., Centurion M., Gühr M., Li R., Liu Y., Moore B., Niebuhr M., Park S., Shen X., Weathersby S., Weinacht T., Martinez T. J., Wang X. (2020). Simultaneous
observation of nuclear and electronic dynamics by ultrafast electron
diffraction. Science.

[33] Yong H., Xu X., Ruddock J. M., Stankus B., Carrascosa A. M., Zotev N., Bellshaw D., Du W., Goff N., Chang Y., Boutet S., Carbajo S., Koglin J. E., Liang M., Robinson J. S., Kirrander A., Minitti M. P., Weber P. M. (2021). Ultrafast X-ray scattering offers
a structural view of excited-state charge transfer. Proc. Nat. Acad. Sci..

[34] Champenois E. G., List N. H., Ware M., Britton M., Bucksbaum P. H., Cheng X., Centurion M., Cryan J. P., Forbes R., Gabalski I., Hegazy K., Hoffmann M. C., Howard A. J., Ji F., Lin M. F., Nunes J. P. F., Shen X., Yang J., Wang X., Martinez T. J., Wolf T. J. (2023). Femtosecond Electronic
and Hydrogen Structural Dynamics in Ammonia Imaged with Ultrafast
Electron Diffraction. Phys. Rev. Lett..

[35] Gabalski I., Green A., Lenzen P., Allum F., Bain M., Bhattacharyya S., Britton M. A., Champenois E. G., Cheng X., Cryan J. P., Driver T., Forbes R., Garratt D., Ghrist A. M., Graßl M., Kling M. F., Larsen K. A., Liang M., Lin M.-F., Liu Y., Minitti M. P., Nelson S., Robinson J. S., Bucksbaum P. H., Wolf T. J. A., List N. H., Glownia J. M. (2025). Imaging valence
electron rearrangement in a chemical reaction using hard X-ray scattering. Phys. Rev. Lett..

[36] Green A. E., Liu Y., Allum F., Graßl M., Lenzen P., Ashfold M. N. R., Bhattacharyya S., Cheng X., Centurion M., Crane S. W., Forbes R., Goff N. A., Huang L., Kaufman B., Kling M.-F., Kramer P. L., Lam H. V. S., Larsen K. A., Lemons R., Lin M.-F., Orr-Ewing A. J., Rolles D., Rudenko A., Saha S. K., Searles J., Shen X., Weathersby S., Weber P. M., Zhao H., Wolf T. J. A. (2025). Imaging the photochemistry
of cyclobutanone using ultrafast
electron diffraction: Experimental results. J. Chem. Phys..

[37] Schalk O., Galiana J., Geng T., Larsson T. L., Thomas R. D., Fdez Galván I., Hansson T., Vacher M. (2020). Competition between
ring-puckering and ring-opening excited state reactions exemplified
on 5H-furan-2-one and derivatives. J. Chem.
Phys..

[38] Merritt I. C., Jacquemin D., Vacher M. (2023). Nonadiabatic Coupling in Trajectory
Surface Hopping: How Approximations Impact Excited-State Reaction
Dynamics. J. Chem. Theory Comput..

[39] Zhao X., Merritt I. C., Lei R., Shu Y., Jacquemin D., Zhang L., Xu X., Vacher M., Truhlar D. G. (2023). Nonadiabatic
Coupling in Trajectory Surface Hopping: Accurate Time Derivative Couplings
by the Curvature-Driven Approximation. J. Chem.
Theory Comput..

[40] Delmas V., Nardi A. N., Merritt I. C., Ferté A., Fdez. Galván I., Vacher M. (2025). Automated Selection
of Nuclear Coordinates
for Reduced Dimensionality Nonadiabatic Dynamics. J. Chem. Theory Comput..

[41] Northey T., Zotev N., Kirrander A. (2014). Ab initio calculation of molecular
diffraction. J. Chem. Theory Comput..

[42] Moreno
Carrascosa A., Yong H., Crittenden D. L., Weber P. M., Kirrander A. (2019). Ab-initio calculation of total x-ray
scattering from molecules. J. Chem. Theory Comput..

[43] Zotev N., Moreno Carrascosa A., Simmermacher M., Kirrander A. (2020). Excited Electronic
States in Total Isotropic Scattering from Molecules. J. Chem. Theory Comput..

[44] International Tables for Crystallography Vol. C: Mathematical, Physical and Chemical Tables, 2006th ed.; Prince, E. , Ed.; Wiley, 2006.

[45] Northey T., Moreno Carrascosa A., Schäfer S., Kirrander A. (2016). Elastic X-ray
scattering from state-selected molecules. J.
Chem. Phys..

[46] Bentley J. J., Stewart R. F. (1975). Total x-ray scattering by H_2_. J. Chem. Phys..

[47] Kohl D. A., Shipsey E. J. (1992). Elastic electron
scattering from state-selected molecules
I. Intensities. Z. Phys. D.

[48] Debnarova A., Techert S., Schmatz S. (2006). *Ab
initio* treatment
of time-resolved x-ray scattering: Application to the photoisomerization
of stilbene. J. Chem. Phys..

[49] Kirrander A. (2012). X-ray diffraction
assisted spectroscopy of Rydberg states. J.
Chem. Phys..

[50] Dixit G., Vendrell O., Santra R. (2012). Imaging electronic quantum motion
with light. Proc. Natl. Acad. Sci..

[51] Moreno
Carrascosa A., Kirrander A. (2017). Ab initio calculation of inelastic
scattering. Phys. Chem. Chem. Phys..

[52] Moreno
Carrascosa A., Northey T., Kirrander A. (2017). Imaging rotations
and vibrations in polyatomic molecules with X-ray scattering. Phys. Chem. Chem. Phys..

[53] Northey T., Kirrander A. (2019). Ab Initio
Fragment Method for Calculating Molecular
X-ray Diffraction. J. Phys. Chem. A.

[54] Simmermacher M., Henriksen N. E., Møller K. B., Moreno Carrascosa A., Kirrander A. (2019). Electronic Coherence in Ultrafast
X-Ray Scattering
from Molecular Wave Packets. Phys. Rev. Lett..

[55] Simmermacher M., Moreno Carrascosa A., Henriksen N. E., Møller K. B., Kirrander A. (2019). Theory of
ultrafast x-ray scattering by molecules in
the gas phase. J. Chem. Phys..

[56] Parrish R. M., Martínez T. J. (2019). Ab Initio Computation of Rotationally-Averaged Pump-Probe
X-ray and Electron Diffraction Signals. J. Chem.
Theory Comput..

[57] Genoni A., Macchi P. (2020). Quantum Crystallography
in the Last Decade: Developments
and Outlooks. Crystals.

[58] Giri S., Tremblay J. C., Dixit G. (2021). Imaging charge migration in chiral
molecules using time-resolved x-ray diffraction. Phys. Rev. A.

[59] Keefer D., Aleotti F., Rouxel J. R., Segatta F., Gu B., Nenov A., Garavelli M., Mukamel S. (2021). Imaging conical intersection
dynamics during azobenzene photoisomerization by ultrafast X-ray diffraction. Proc. Natl. Acad. Sci..

[60] Lingerfelt D. B., Yoshimura A., Jakowski J., Ganesh P., Sumpter B. G. (2022). Extracting
Inelastic Scattering Cross Sections for Finite and Aperiodic Materials
from Electronic Dynamics Simulations. J. Chem.
Theory Comput..

[61] Moreno
Carrascosa A., Coe J. P., Simmermacher M., Paterson M. J., Kirrander A. (2022). Towards high-resolution X-ray scattering
as a probe of electron correlation. Phys. Chem.
Chem. Phys..

[62] Ziems K. M., Simmermacher M., Gräfe S., Kirrander A. (2023). The contribution
of Compton ionization to ultrafast x-ray scattering. J. Chem. Phys..

[63] Liane E. M., Simmermacher M., Kirrander A. (2024). Ultrafast x-ray scattering and electronic
coherence at avoided crossings: complete isotropic signals. J. Phys. B:At. Mol. Opt. Phys..

[64] Kirrander A., Saita K., Shalashilin D. V. (2016). Ultrafast
X-ray Scattering from Molecules. J. Chem. Theory
Comput..

[65] Kirrander A., Weber P. M. (2017). Fundamental Limits
on Spatial Resolution in Ultrafast
X-ray Diffraction. Appl. Science.

[66] Martinez T. J., Ben-Nun M., Levine R. D. (1996). Multi-Electronic-State Molecular
Dynamics: A Wave Function Approach with Applications. J. Phys. Chem..

[67] Ben-Nun M., Quenneville J., Martínez T. J. (2000). Ab Initio Multiple Spawning: Photochemistry
from First Principles Quantum Molecular Dynamics. J. Phys. Chem. A.

[68] Curchod, B. F. E. In Quantum Chemistry and Dynamics of Excited States: Methods and Applications, 1st ed.; González, L. , Lindh, R. , Eds.; John Wiley and Sons: UK, 2020; Chapter 14, p 435.

[69] Kirrander, A. ; Vacher, M. In Quantum Chemistry and Dynamics of Excited States: Methods and Applications, 1st ed.; González, L. , Lindh, R. , Eds.; John Wiley and Sons: UK, 2020; Chapter 15, p 469.

[70] Richings G., Polyak I., Spinlove K., Worth G., Burghardt I., Lasorne B. (2015). Quantum dynamics simulations
using Gaussian wavepackets:
the vMCG method. Int. Rev. Phys. Chem..

[71] Worth, G. A. ; Lasorne, B. In Quantum Chemistry and Dynamics of Excited States: Methods and Applications, 1st ed.; González, L. , Lindh, R. , Eds.; John Wiley and Sons: UK, 2020; Chapter 13, p 413.

[72] Tully J. C. (1990). Molecular
dynamics with electronic transitions. J. Chem.
Phys..

[73] Mai, S. ; Marquetand, P. ; González, L. In Quantum Chemistry and Dynamics of Excited States: Methods and Applications, 1st ed.; González, L. , Lindh, R. , Eds.; John Wiley and Sons: UK, 2020; Chapter 16, p 499.

[74] Crespo-Otero R., Barbatti M. (2018). Recent Advances and
Perspectives on Nonadiabatic Mixed
Quantum–Classical Dynamics. Chem. Rev..

[75] Finley J., Malmqvist P.-Å., Roos B. O., Serrano-Andrés L. (1998). The multi-state
CASPT2 method. Chem. Phys. Lett..

[76] Granovsky A. A. (2011). Extended
multi-configuration quasi-degenerate perturbation theory: The new
approach to multi-state multi-reference perturbation theory. J. Chem. Phys..

[77] Roos B. O., Taylor P. R., Sigbahn P. E. (1980). A complete
active space SCF method
(CASSCF) using a density matrix formulated super-CI approach. Chem. Phys..

[78] Runge E., Gross E. K. U. (1984). Density-Functional
Theory for Time-Dependent Systems. Phys. Rev.
Lett..

[79] Janoš J., Slavíček P. (2023). What Controls
the Quality of Photodynamical
Simulations? Electronic Structure Versus Nonadiabatic Algorithm. J. Chem. Theory Comput..

[80] Papineau T. V., Jacquemin D., Vacher M. (2024). Which Electronic Structure Method
to Choose in Trajectory Surface Hopping Dynamics Simulations? Azomethane
as a Case Study. J. Phys. Chem. Lett..

[81] Cooper J. C., Brown C. Y. Z., Kára J., Kirrander A. (2025). Photoexcited
dynamics of the valence states of norbornadiene. J. Chem. Phys..

[82] Bellshaw D., Minns R. S., Kirrander A. (2019). Correspondence
between electronic
structure calculations and simulations: nonadiabatic dynamics in CS_2_. Phys. Chem. Chem. Phys..

[83] Cigrang L. L. E., Curchod B. F. E., Ingle R. A., Kelly A., Mannouch J. R., Accomasso D., Alijah A., Barbatti M., Chebbi W., Došlić N., Eklund E. C., Fernandez-Alberti S., Freibert A., González L., Granucci G., Hernández F. J., Hernández-Rodríguez J., Jain A., Janoš J., Kassal I., Kirrander A., Lan Z., Larsson H. R., Lauvergnat D., Le Dé B., Lee Y., Maitra N. T., Min S. K., Peláez D., Picconi D., Qiu Z., Raucci U., Robertson P., Sangiogo Gil E., Sapunar M., Schürger P., Sinnott P., Tretiak S., Tikku A., Vindel-Zandbergen P., Worth G. A., Agostini F., Gómez S., Ibele L. M., Prlj A. (2025). Roadmap for Molecular Benchmarks
in Nonadiabatic Dynamics. J. Phys. Chem. A.

[84] Prlj A., Taylor J. T., Janoš J., Lognon E., Hollas D., Slavíček P., Agostini F., Curchod B. F. E. (2026). Best
practices for nonadiabatic molecular dynamics simulations. Living J. Comp. Mol. Sci..

[85] Polyak I., Hutton L., Crespo-Otero R., Barbatti M., Knowles P. J. (2019). Ultrafast
Photoinduced Dynamics of 1,3-Cyclohexadiene Using XMS-CASPT2 Surface
Hopping. J. Chem. Theory Comput..

[86] Razmus W. O., Acheson K., Bucksbaum P., Centurion M., Champenois E., Gabalski I., Hoffman M. C., Howard A., Lin M. F., Liu Y., Nunes P., Saha S., Shen X., Ware M., Warne E. M., Weinacht T., Wilkin K., Yang J., Wolf T. J., Kirrander A., Minns R. S., Forbes R. (2022). Multichannel photodissociation
dynamics
in CS2 studied by ultrafast electron diffraction. Phys. Chem. Chem. Phys..

[87] Bertram L., Weber P., Kirrander A. (2023). Mapping the
photochemistry of cyclopentadiene:
from theory to ultrafast X-ray scattering. Faraday
Discuss..

[88] Aquilante F., Autschbach J., Baiardi A., Battaglia S., Borin V. A., Chibotaru L. F., Conti I., De Vico L., Delcey M., Fdez. Galván I., Ferré N., Freitag L., Garavelli M., Gong X., Knecht S., Larsson E. D., Lindh R., Lundberg M., Malmqvist P. A. ˙., Nenov A., Norell J., Odelius M., Olivucci M., Pedersen T. B., Pedraza-González L., Phung Q. M., Pierloot K., Reiher M., Schapiro I., Segarra-Martí J., Segatta F., Seijo L., Sen S., Sergentu D.-C., Stein C. J., Ungur L., Vacher M., Valentini A., Veryazov V. (2020). Modern quantum chemistry with [Open]­Molcas. J. Chem. Phys..

[89] Folkestad S. D., Kjønstad E. F., Myhre R. H., Andersen J. H., Balbi A., Coriani S., Giovannini T., Goletto L., Haugland T. S., Hutcheson A., Høyvik I.-M., Moitra T., Paul A. C., Scavino M., Skeidsvoll A. S., Tveten Å. H., Koch H. (2020). eT 1.0: An open source
electronic structure program with emphasis
on coupled cluster and multilevel methods. J.
Chem. Phys..

[90] Werner, H.-J. ; Knowles, P. J. ; Knizia, G. ; Manby, F. R. ; Schütz, M. ; MOLPRO, Version 2012.1; National Institutes of Natural Sciences, 2012..

[91] Wang J., Smith V. H. (1994). Evaluation of cross sections for X-ray and high-energy
electron scattering from molecular systems. Int. J. Quantum Chem..

[92] Sun Q., Berkelbach T. C., Blunt N. S., Booth G. H., Guo S., Li Z., Liu J., McClain J. D., Sayfutyarova E. R., Sharma S., Wouters S., Chan G. K.-L. (2018). PySCF: the Python-based
simulations of chemistry framework. WIREs Comput.
Mol. Sci..

[93] Mai, S. ; Avagliano, D. ; Heindl, M. ; Marquetand, P. ; Menger, M. F. S. J. ; Oppel, M. ; Plasser, F. ; Polonius, S. ; Ruckenbauer, M. ; Shu, Y. ; Truhlar, D. G. ; Zhang, L. ; Zobel, P. ; González, L. SHARC3.0: Surface Hopping Including Arbitrary Couplings– Program Package for Non-Adiabatic Dynamics, Version v2.1.2; sharc-md.org, 2023..

[94] Granucci G., Persico M., Zoccante A. (2010). Including
quantum decoherence in
surface hopping. J. Chem. Phys..

[95] Suominen H. J., Kirrander A. (2014). How to observe coherent electron dynamics directly. Phys. Rev. Lett..

[96] Northey T., Kirrander A., Weber P. M. (2024). Extracting the electronic structure
signal from X-ray and electron scattering in the gas phase. J. Synchrotron Rad..

[97] Yang J., Makhija V., Kumarappan V., Centurion M. (2014). Reconstruction
of three-dimensional molecular structure from diffraction of laser-aligned
molecules. Struct. Dyn..

[98] Minitti M.
P., Budarz J. M., Kirrander A., Robinson J., Lane T. J., Ratner D., Saita K., Northey T., Stankus B., Cofer-Shabica V., Hastings J., Weber P. M. (2014). Toward structural
femtosecond chemical dynamics: imaging chemistry in space and time. Faraday Discuss..

[99] Yong H., Ruddock J. M., Stankus B., Ma L., Du W., Goff N., Chang Y., Zotev N., Bellshaw D., Boutet S., Carbajo S., Koglin J. E., Liang M., Robinson J. S., Kirrander A., Minitti M. P., Weber P. M. (2019). Scattering
off molecules far from equilibrium. J. Chem.
Phys..

[100] Yong H., Moreno Carrascosa A., Ma L., Stankus B., Minitti M. P., Kirrander A., Weber P. M. (2021). Determination of
excited state molecular structures from time-resolved gas-phase X-ray
scattering. Faraday Discuss..

[101] Acheson K., Kirrander A. (2023). Robust Inversion of Time-Resolved
Data via Forward-Optimization in a Trajectory Basis. J. Chem. Theory Comput..

[102] Odate A., Kirrander A., Weber P. M., Minitti M. P. (2023). Brighter,
faster, stronger: ultrafast scattering of free molecules. Adv. Phys. X.

[103] Pemberton C. C., Zhang Y., Saita K., Kirrander A., Weber P. M. (2015). From the (1B) Spectroscopic State
to the Photochemical
Product of the Ultrafast Ring-Opening of 1,3-Cyclohexadiene: A Spectral
Observation of the Complete Reaction Path. J.
Phys. Chem. A.

[104] Tudorovskaya M., Minns R. S., Kirrander A. (2018). Effects of
probe energy and competing pathways on time-resolved photoelectron
spectroscopy: the ring-opening of 1,3-cyclohexadiene. Phys. Chem. Chem. Phys..

[105] Acheson K., Kirrander A. (2023). Automatic Clustering of Excited-State
Trajectories: Application to Photoexcited Dynamics. J. Chem. Theory Comput..

[106] Kára J., Acheson K., Kirrander A. (2025). DONKEY: A
Flexible and Accurate Algorithm for Clustering. J. Chem. Theory Comput..

[107] Janoš, J. ; List, N. H. ; Orr-Ewing, A. J. ; Suchan, J. ; Barbatti, M. ; Bennett, O. ; Brady, M. ; Carmona-García, J. ; Crespo-Otero, R. ; Eng, J. ; Fajen, O. J. ; Garavelli, M. ; Gómez, S. ; Green, A. E. ; Hernández, F. J. ; Hollas, D. ; Hutton, L. ; Ibele, L. M. ; Kirrander, A. ; Lan, Z. ; Lassmann, Y. ; Lawrence, J. E. ; Levine, B. G. ; Makhov, D. V. ; Mannouch, J. R. ; Miao, X. ; Mitrić, R. ; Parker, S. M. ; Penfold, T. J. ; Peng, J. ; Richardson, J. O. ; Shalashilin, D. ; Slavíček, P. ; Spinlove, K. E. ; Vindel-Zandbergen, P. ; Agostini, F. ; Bonella, S. ; Martínez, T. J. ; Worth, G. A. ; Curchod, B. F. E. Perspective on a challenge: predicting the photochemistry of cyclobutanone. https://arxiv.org/abs/2604.12749 (accessed May 2026). 10.1063/5.0338792 42446072

[108] Borne K.
D., Cooper J. C., Ashfold M. N. R., Bachmann J., Bhattacharyya S., Boll R., Bonanomi M., Bosch M., Callegari C., Centurion M., Coreno M., Curchod B. F. E., Danailov M. B., Demidovich A., Di Fraia M., Erk B., Faccialà D., Feifel R., Forbes R. J. G., Hansen C. S., Holland D. M. P., Ingle R. A., Lindh R., Ma L., McGhee H. G., Muvva S. B., Nunes J. P. F., Odate A., Pathak S., Plekan O., Prince K. C., Rebernik P., Rouzée A., Rudenko A., Simoncig A., Squibb R. J., Venkatachalam A. S., Vozzi C., Weber P. M., Kirrander A., Rolles D. (2024). Ultrafast electronic relaxation pathways of the molecular
photoswitch quadricyclane. Nat. Chem..

[109] Garrow M., Bertram L., Winter A., Prentice A. W., Crane S. W., Lane P. D., Greaves S. J., Paterson M. J., Kirrander A., Townsend D. (2025). Excited state dynamics
of azanaphthalenes
reveal opportunities for the rational design of photoactive molecules. Commun. Chem..

[110] Penfold T. J., Curchod B. F. (2024). Exploring the Influence
of Approximations
for Simulating Valence Excited X-ray Spectra. J. Phys. Chem. A.

[111] Ruddock J. M., Zotev N., Stankus B., Yong H.-W., Bellshaw D., Boutet S., Lane T. J., Liang M., Carbajo S., Du W., Kirrander A., Minitti M. P., Weber P. M. (2019). Simplicity beneath Complexity: Counting
Molecular Electrons Reveals Transients and Kinetics of Photodissociation
Reactions. Angew. Chem., Int. Ed..

