# Supplementary material for: The Importance of Going beyond the Independent Atom Model When Predicting UED Signals from Simulations
Source: J Chem Theory Comput. 2026 Jun 10;22(12):5945–56. doi: 10.1021/acs.jctc.6c00207 (PMC13296531; doi:10.1021/acs.jctc.6c00207)
Supplement: Supplementary file 1 [file ct6c00207_si_001.pdf]

Supplementary Information
The importance of going beyond the independent atom model when predicting UED signals from simulations

Lewis Hutton,*,†,‡ Andrés Moreno Carrascosa,† Mats Simmermacher,† and Adam Kirrander*,†

†*Physical and Theoretical Chemistry Laboratory, Department of Chemistry, University of Oxford, Oxford OX1 3QZ, United Kingdom*

‡*Current address: Instituto de Ciencia Molecular, Universitat de València, Apartado22085, ES-46071 Valencia, Spain*

E-mail: lewis.hutton@uv.es; adam.kirrander@chem.ox.ac.uk

Phone: +44 (0)1865 275400

Active spaces

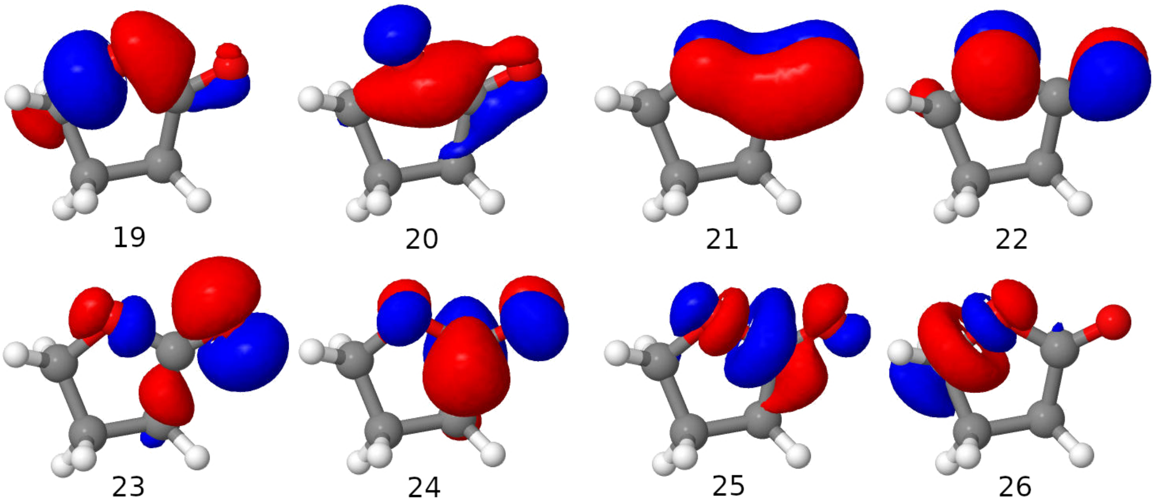

Figure S1: Active space consisting of 10 electrons in 8 orbitals employed for SA-CASSCF and XMS-CASPT2 calculations. The active space is made of 2 σ, 2 π, 1 n, 1 π^* and 2 σ^* orbitals.

Table S1: A summary of the orbitals used for each active space. A ✓indicates the orbital is included in the active space whereas - shows the orbital is absent. Orbital number correspond to those given in Fig. S1.

Active space	σ (19)	σ (20)	π (21)	π (22)	n (23)	π^* (24)	σ^* (25)	σ^* (26)
(10,8)	✓	✓	✓	✓	✓	✓	✓	✓
(8,7)	✓	-	✓	✓	✓	✓	✓	✓
(8,6)	-	✓	✓	✓	✓	✓	-	✓
(6,5)	-	-	✓	✓	✓	✓	-	✓
(6,4)	-	-	✓	✓	✓	✓	-	-
(4,3)	-	-	-	✓	✓	✓	-	-

Effect of sampling on the pump-off $t < 0$ reference signal

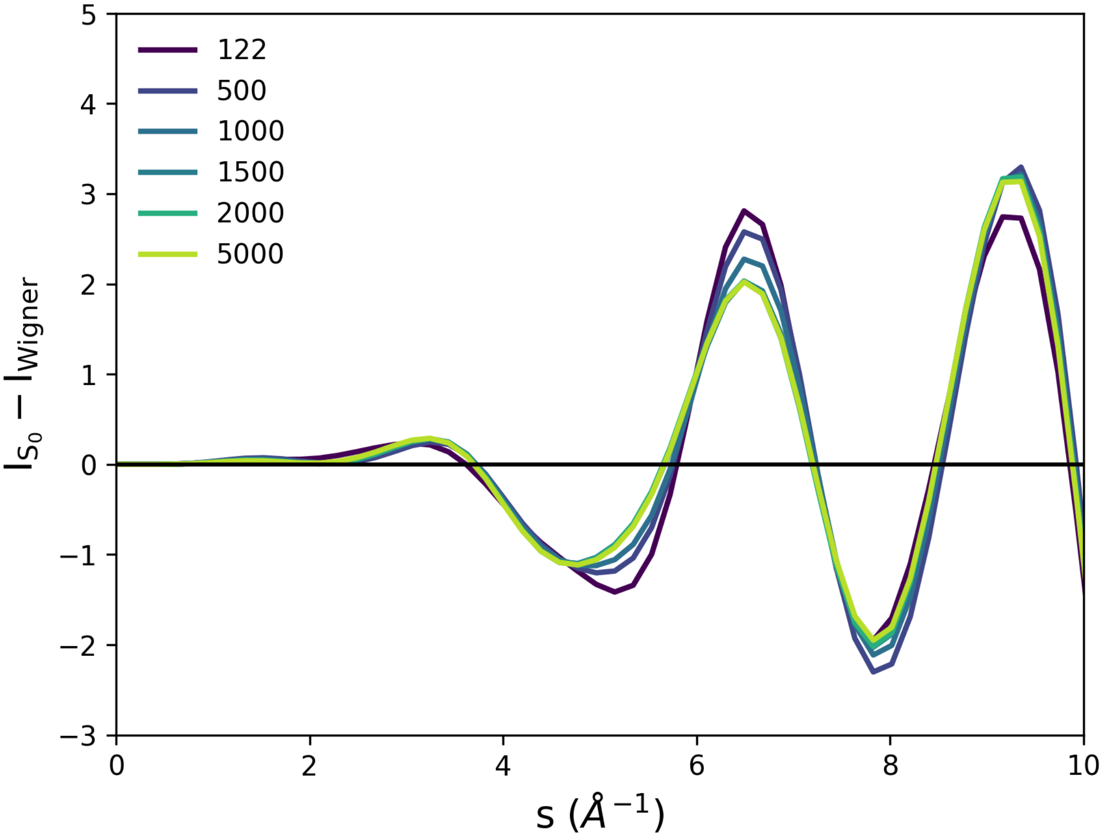

Figure S2: Changes in the scattering for the pump-off $t < 0$ fs reference signal as more and more molecular geometries from the equilibrium ground state Wigner distribution are included. The figure shows the difference signal, $I_{S_0} - I_{Wigner}$, taken as a difference between the single equilibrium geometry, $I_{S_0}(s)$, and the average Wigner sampled signal, I_{Wigner}. The latter is shown for the 122 geometries used to initiate the trajectories, as well as separate Wigner-sampled distributions with 500, 1000, 1500, 2000, and 5000 geometries.

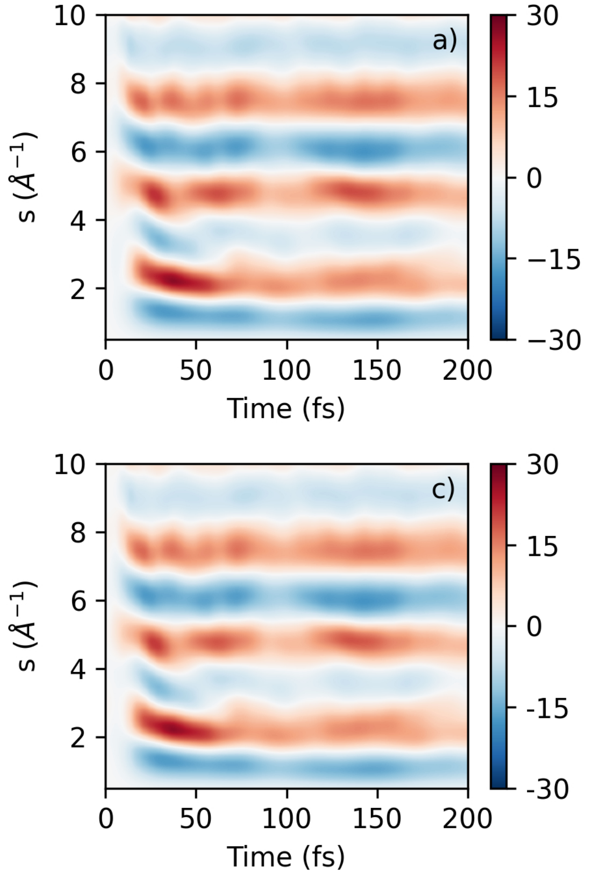

Figure S3: The time-dependent percent difference signal, $\%\triangle I_{ref}(s)$, calculated using three different reference pump-off $t < 0$ signals, $I_{ref}(s)$. In panel a) we show the results when only the S_0 minimum energy geometry $I_1(s)$ is considered as the reference, in panel b) all the 122 molecular geometries used as initial conditions for the trajectories in the TSH simulations are used $I_{122}(s)$, and finally in c) 1000 Wigner-sampled geometries are used $I_{1000}(s)$.

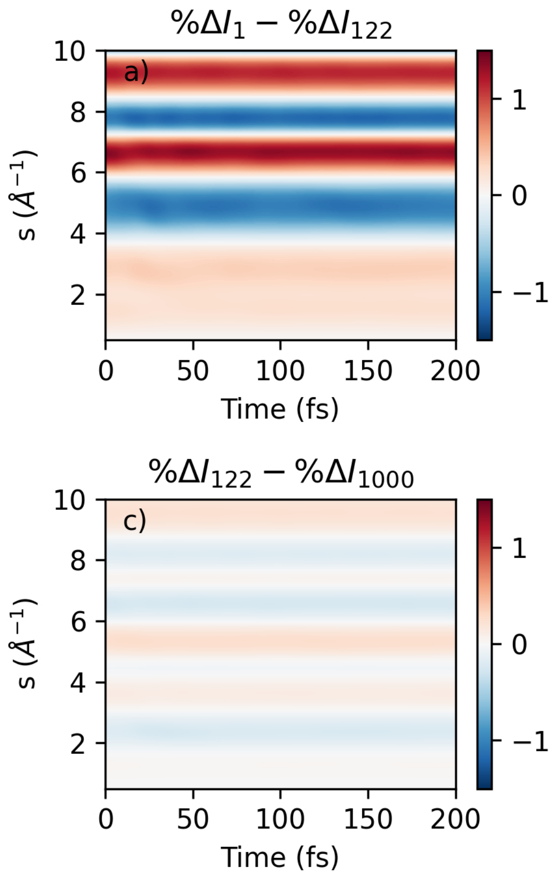

Figure S4: The differences between the percent difference signals shown in Fig. S3, showing how large the changes in the percent difference signal are depending on which pump-off reference signal is used. The change in going from a single reference geometry, $I_1(s)$, is significant (approximately 1%) but the difference between using 122 or 1000 structures to calculate the reference signal are rather small (as can be anticipated from Fig. S2).

Electronic structure benchmarking

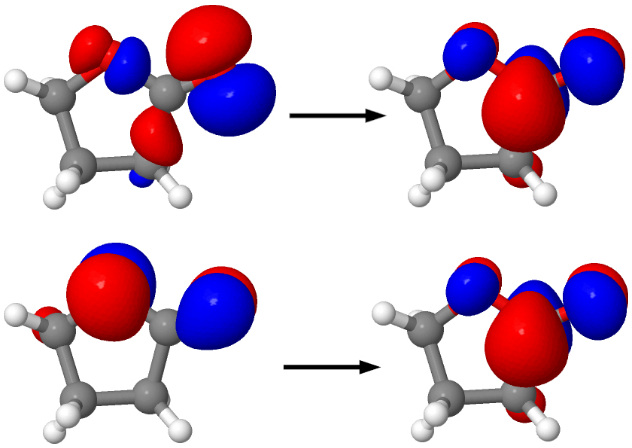

Figure S5: The two lowest electronic transitions in γ-butyrolactone at the S_0 equilibrium geometry, corresponding to the two lowest-lying singlet states. The transition at the top of the figure shows the dark S_1 nπ^* state and the bottom transition shows the bright S_2 $\pi\pi^*$ state.

A summary of the low-lying excited singlet states of γ-butyrolacetone is given in Table S2. The three electronic structure methods SA(3)-CASSCF(10,8), XMS(3)-CASPT2(10,8), and CC3 yield the same state ordering, with the S_1 state having nπ^* character and S_2 $\pi\pi^*$ character. Both transitions are shown in Fig. SI S5. According to CC3, the dark S_1 state has an excitation energy of 6.01 eV at the equilibrium geometry, with SA(3)-CASSCF(10,8) and XMS(3)-CASPT2(10,8) in close agreement, under and overestimating the excitation energy by 0.07 and 0.10 eV, respectively. However, larger discrepancies are observed for the bright S_2 $\pi\pi^*$ state where SA(3)-CASSCF(10,8) overestimates the energy by 0.91 eV compared to CC3. For XMS(3)-CASPT2, the S_2 excitation energy is closer to the CC3 reference, although still too high by 0.17 eV. This indicates that dynamic correlation is important in the S_2 $\pi\pi^*$ state. Notably, the excitation energies found here are consistent with previous results (SA(3)-CASSCF(10,8)/ANO-RCC-VDZP).[1]

Table S2: Excitation energies and state characters (in parenthesis) for the two lowest-lying singlet excited states, S_1 and S_2, in γ-butyrolacetone at the SA(3)-CASSCF(10,8)/cc-pVDZ equilibrium geometry. Results for SA-CASSCF(10,8), XMS-CASPT2 and CC3 are shown.

Method	S_1 excitation energy (eV)	S_2 excitation energy (eV)
SA(3)-CASSCF(10,8)	5.94 ($n\pi^*$)	9.16 ($\pi\pi^*$)
XMS(3)-CASPT2(10,8)	6.11 ($n\pi^*$)	8.42 ($\pi\pi^*$)
CC3	6.01 ($n\pi^*$)	8.25 ($\pi\pi^*$)

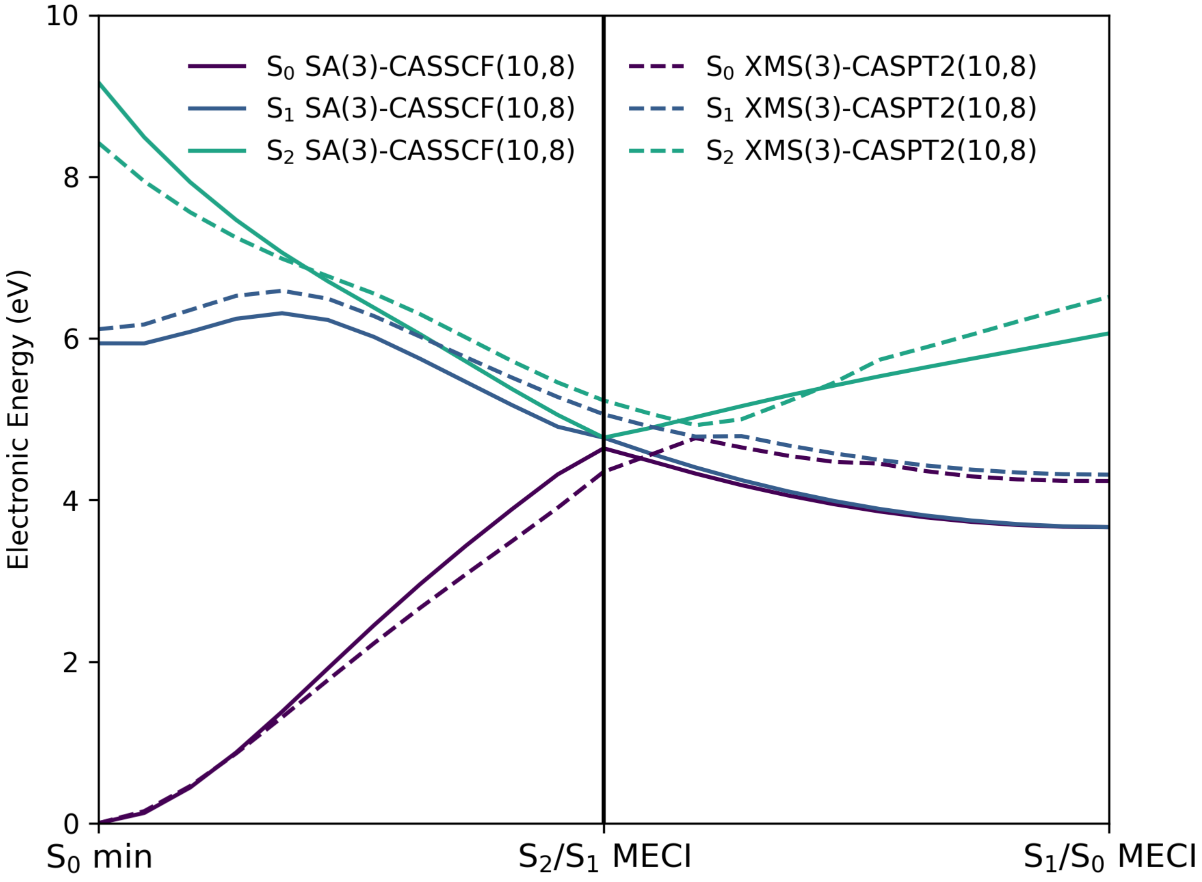

Figure S6: LIIC between the S_0 ground state minimum energy geometry, the S_2/S_1 MECI and the S_1/S_0 MECI. Electronic energies are calculated using SA(3)-CASSCF(10,8)/cc-pVDZ (solid) and XMS(3)-CASPT2(10,8)/cc-pVDZ (dashed) for the three lowest lying singlet states.

Figures SI S6 and S7 show the electronic energies along the LIIC between the S_0 and S_2/S_1 MECI (Fig. SI S7) and onto the S_1/S_2 MECI (Fig. SI S6). Electronic energies are calculated using SA(3)-CASSCF(10,8), XMS(3)-CASPT2(10,8), EOM-CC3, and EOM-CC2.

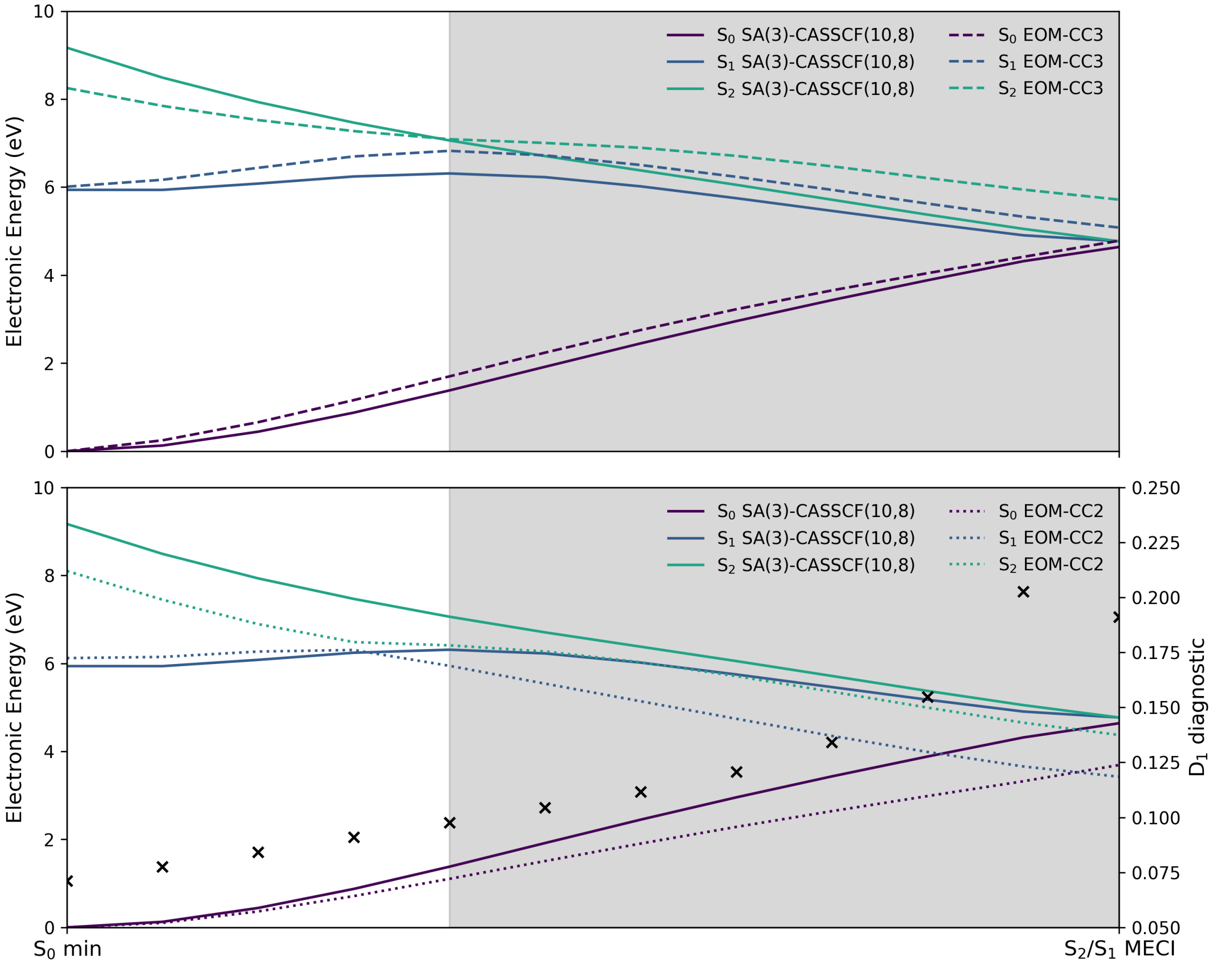

Figure S7: LIICs between the S_0 minimum energy geometry and the S_2/S_1 MECI using SA(3)-CASSCF(10,8) against EOM-CC3 (top panel) and EOM-CC2 (bottom panel) with cc-pVDZ. The bottom panel also shows the D_1 diagnostic (crosses) obtained from the EOM-CC2 calculations. Regions of the LIIC that have a D_1 diagnostic above 0.1 are highlighted with a gray background.

Here, EOM-CC2 calculations were obtained using TURBOMOLE.[2] Figure SI S7 includes the D_1 diagnostic (represented as crosses), which provides a measure of the multireference character of the ground state, to assist in the assessment of the reliability of the CC calculations, with D_1 values above 0.1 indicated by gray background.[3] Overall, all methods agree that the S_2 state is strongly dissociative towards the S_2/S_1 MECI, and XMS(3)-CASPT2(10,8) and SA(3)-CASSCF(10,8) indicate a period of close degeneracy post-S_2/S_1 MECI of the S_1 state and the ground state.

Example trajectories

Quantum yields

Table S3: Quantum yields of three possible outcomes from TSH of γ-butyrolacetone, along with the standard deviation calculated according to Ref. 4.

Process	QY (%)	Error (%)
Ring-opening	82.8	3.4
CO production	17.2	3.4
FC geometry	0.0	0.0

Inspection of the failed trajectories indicates that trajectory-terminating discontinuities in the total energy are most likely to occur during CO dissociation. The QYs reported here are therefore subject to a not insignificant bias from the removal of such trajectories. However, as the calculated UED signals are all calculated on the basis of the same set of trajectories, the comparison between AIS and IAM signals remains valid.

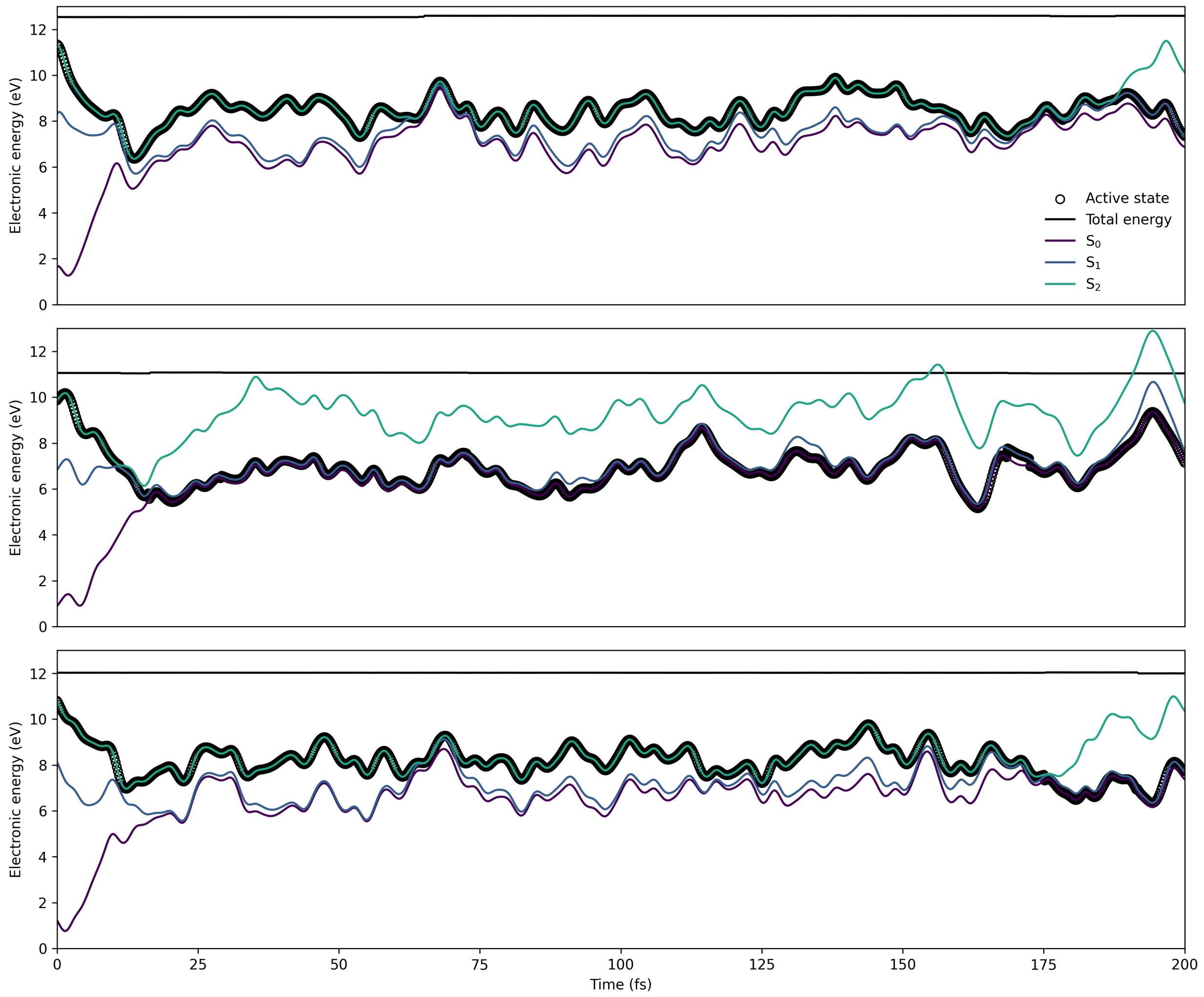

Figure S8: Three exemplary trajectories taking from the TSH simulations showing the three singlet states included, S_0 (purple), S_1 (blue) and S_2 (green). The active state is indicated with a black circle and the total energy with a solid black line.

Active spaces

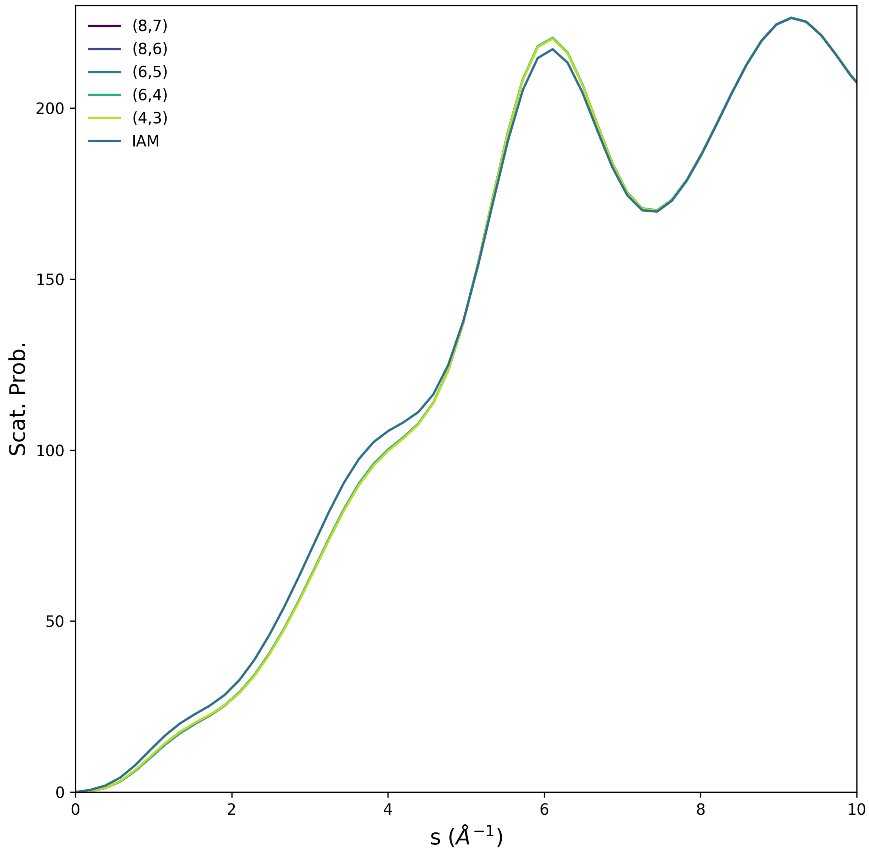

Figure S9: Absolute scattering probabilities for SA(3)-CASSCF with all active spaces in Table S1 and the IAM.

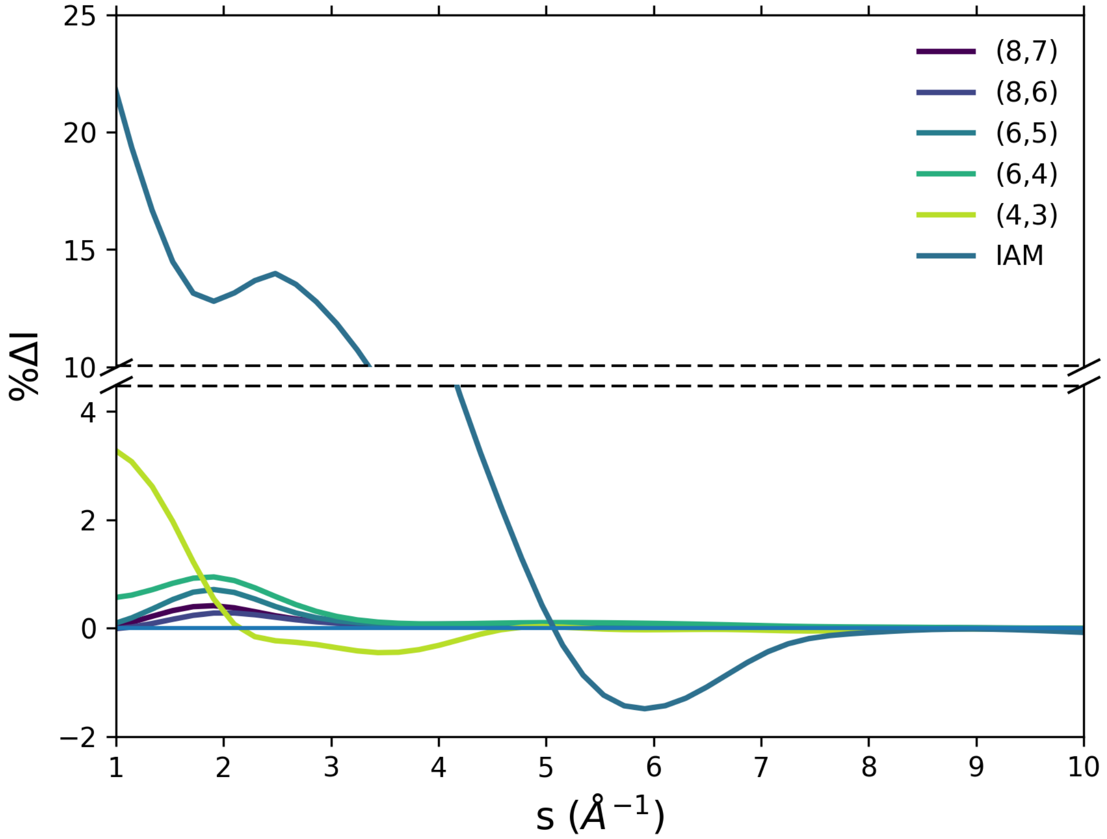

Figure S10: UED signals calculated using the active spaces in Table S1 and the IAM. SA(3)-CASSCF(10,8) is used as the reference and scattering is calculated for the ground electronic state.

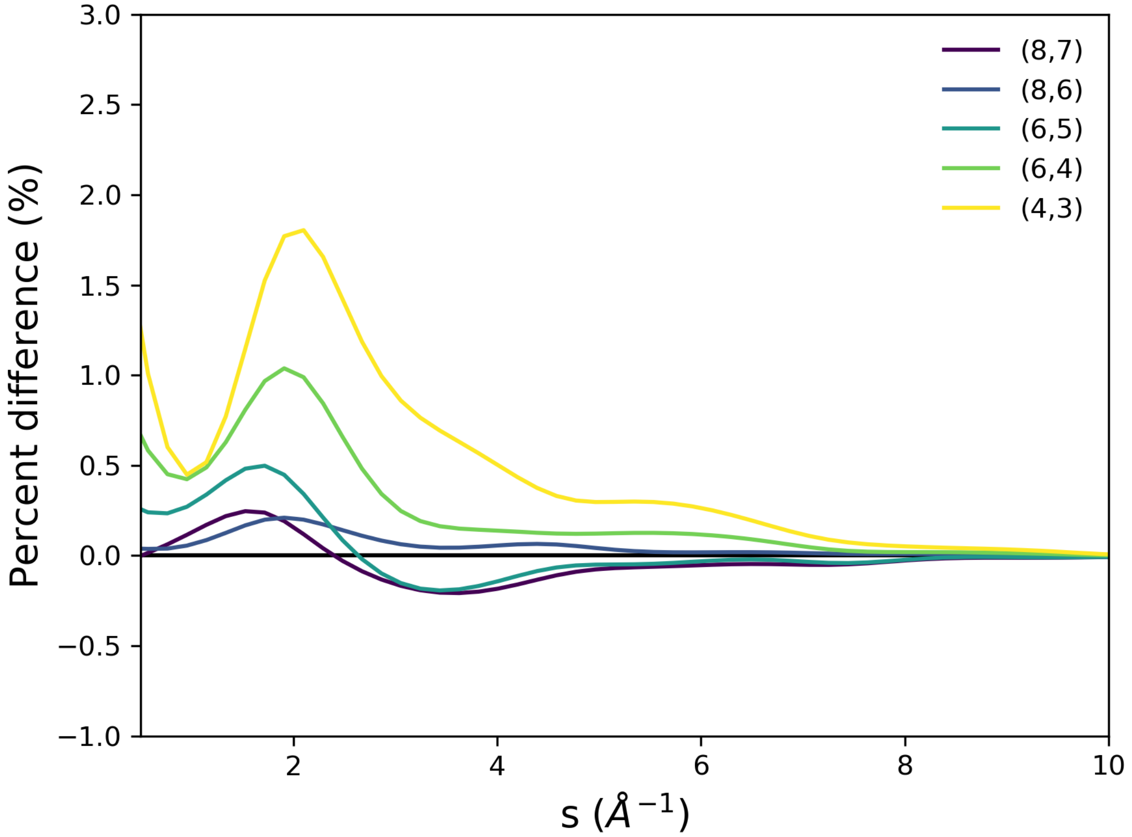

Figure S11: Static UED signals from the S_1 state using SA(3)-CASSCF(10,8) as a reference for the active spaces described in Table S1.

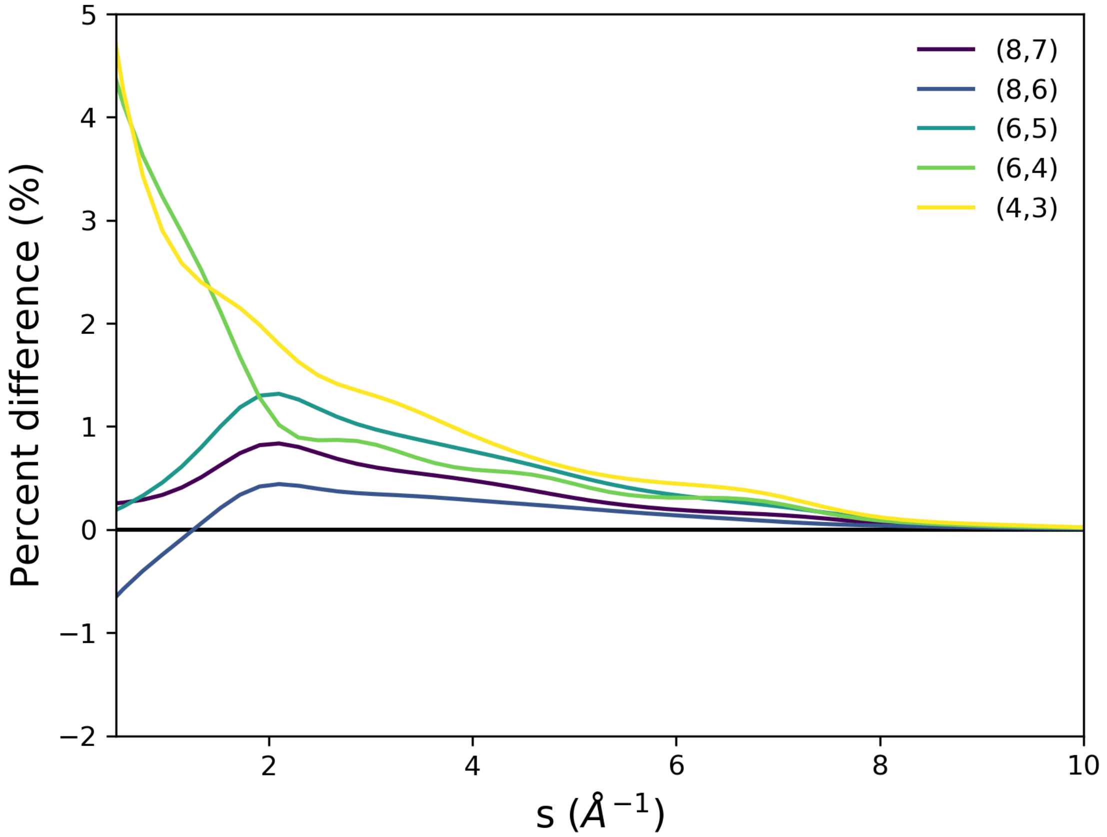

Figure S12: Static UED signals from the S_2 state using SA(3)-CASSCF(10,8) as a reference for the active spaces described in Table S1.

Basis sets

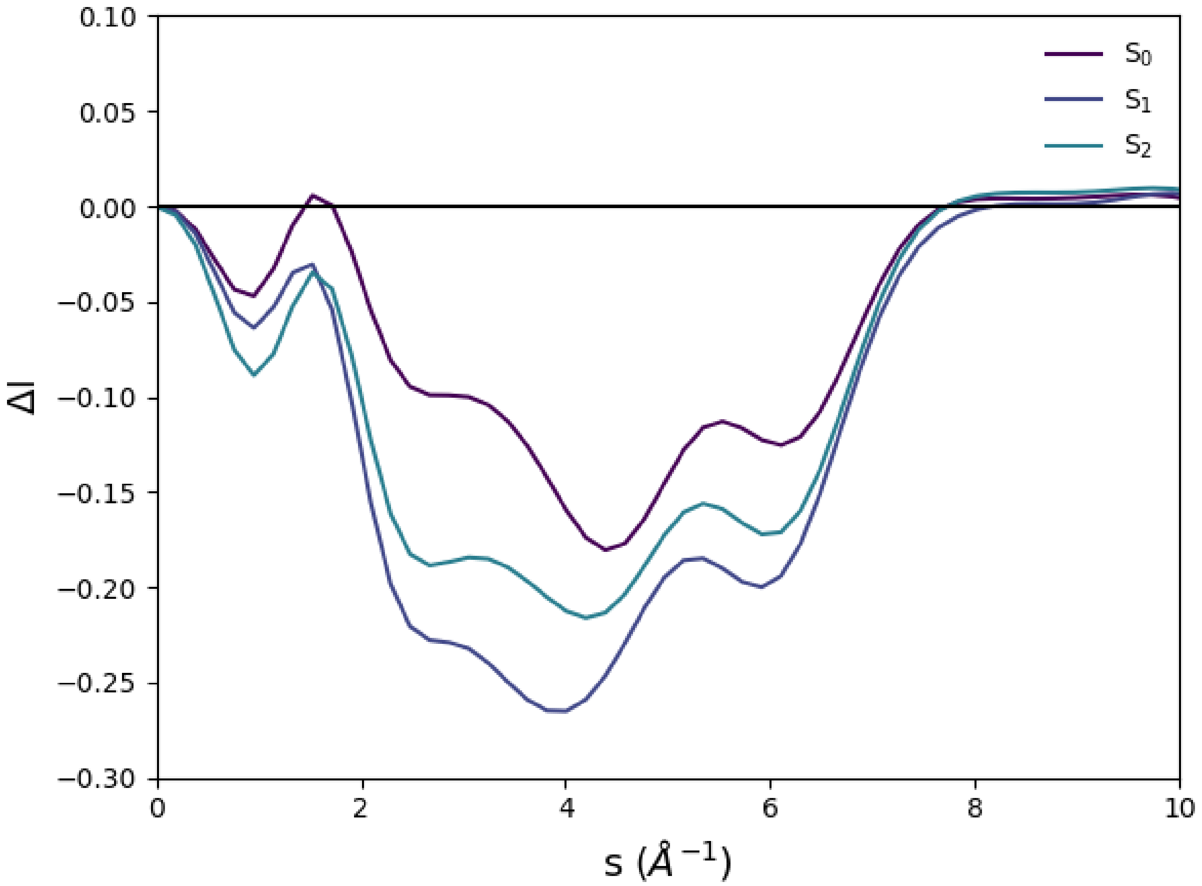

Figure S13: ΔI of the diffraction signals obtained from the AIS in the S_0, S_1 and S_2 states relative to the IAM.

UED along the LIIC

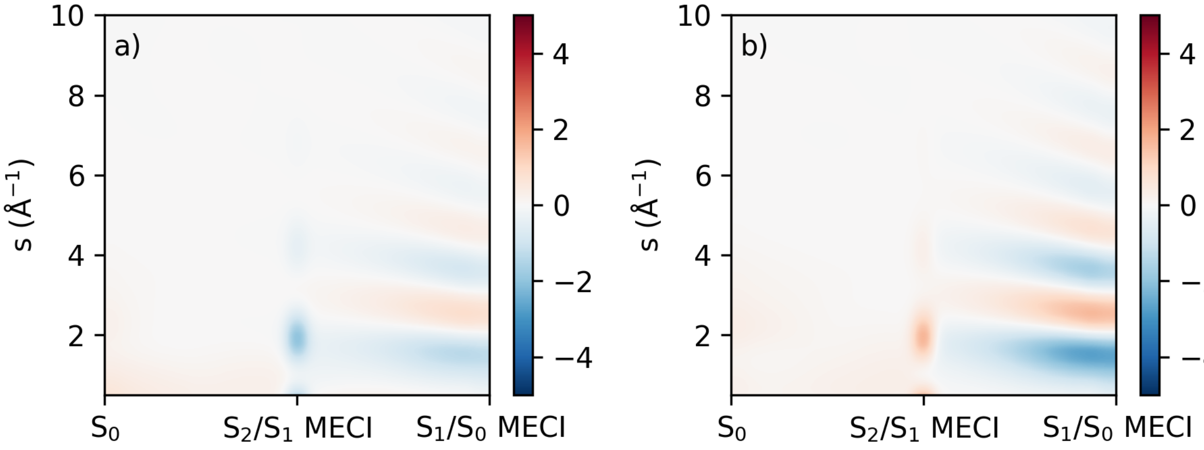

Figure S14: Panel a) shows the $\%\Delta\Delta I(s)$ between UED calculated using SA(3)-CASSCF(10,8)/cc-pVDZ and SA(3)-CASSCF(10,8)/aug-cc-pVDZ for the S_2 state. Panel b) shows $\%\Delta\Delta I(s)$ from the S_1 states.

To determine the effect of including more diffuse basis functions on the UED signal, we have calculated the diffraction signal along the LIIC using aug-cc-pVDZ, shown in Fig. SI S14. Differences of less than 1% are observed until the S_2/S_1 MECI, whereupon errors increase to a maximum of 4% for the S_1 state at the S_1/S_0 MECI. This indicates that it is important to use a diffuse basis when calculating scattering from excited states, although there will invariably be practical limitations linked to the rapid increase in computational cost. As an aside, we note that both panels in Fig. SI S14 show a discontinuity at the S_2/S_1 MECI as a result of a small perturbation from changing the basis set compared to the level of electronic structure theory used to optimize the MECI geometry. The strict energy convergence criterion results in a change of state ordering.

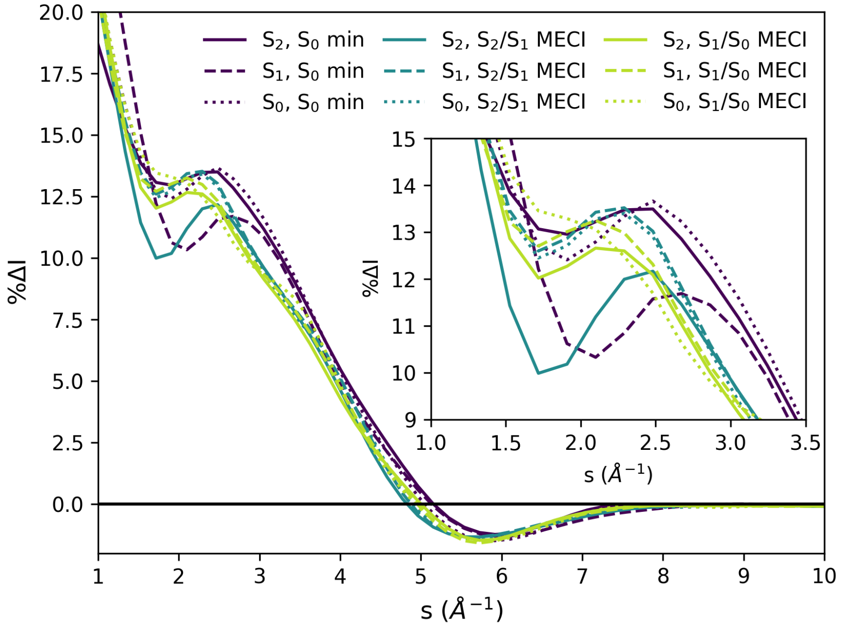

Figure S15: Percent difference total isotropic scattering $\%\Delta I(s)$, as a function of momentum transfer s for γ-butyrolactone at three molecular geometries that define the LIIC: S_0 min equilibrium geometry (purple), S_2/S_1 MECI (blue), and S_1/S_0 MECI (green). At each geometry, the signal is calculated for the S_0 (dotted), S_1 (dashed), and S_2 (solid) electronic states. The percent difference signal is calculated with the IAM taken as $I_{\text{method}}(s, \overline{\mathbf{R}})$ and the AIS signal for each electronic state as the reference $I_{\text{ref}}(s, \overline{\mathbf{R}})$. The AIS calculations are carried out using SA(3)-CASSCF(10,8)/cc-pVDZ. The inset shows a magnified section for $1 < s < 3.5$ Å^{-1}.

The percent difference of the IAM against the SA(3)-CASSCF(10,8)/cc-pVDZ reference, taken for the three electronic states, is shown in Fig. SI S15. The percent differences are calculated at the three characteristic molecular geometries used to defined the LIIC, namely the equilibrium geometry (S_0 min), and the two MECIs (S_2/S_1 and S_1/S_0 MECI). This helps us examine the systematic error in IAM compared to AIS. The percent differences are not very sensitive to molecular geometry, with any such dependence relating to the fact that a C−O bond is broken as we go from the equilibrium to the two MECIs. This geometry effect is most visible between 3 and 5 Å^{-1}, and the signals approximately group according to geometry in this range (each molecular geometry corresponding to a different line color). In contrast, in the s range of $1.5 < s < 2.5$ Å^{-1}, the nature of the electronic state is more

important than molecular geometry. We also note that the results shown in Fig. 4 (main text) indicate that the scattering from different electronic states can differ by some ±5%; it is clear from Fig. SI S15 that the overall differences to IAM are much greater, approaching 15% irrespective of molecular geometry and electronic state considered. There is also, according to Fig. S15, a systematic underestimate of the signal at larger values of s, in the range $5 < s < 7.5$ Å^{-1}.

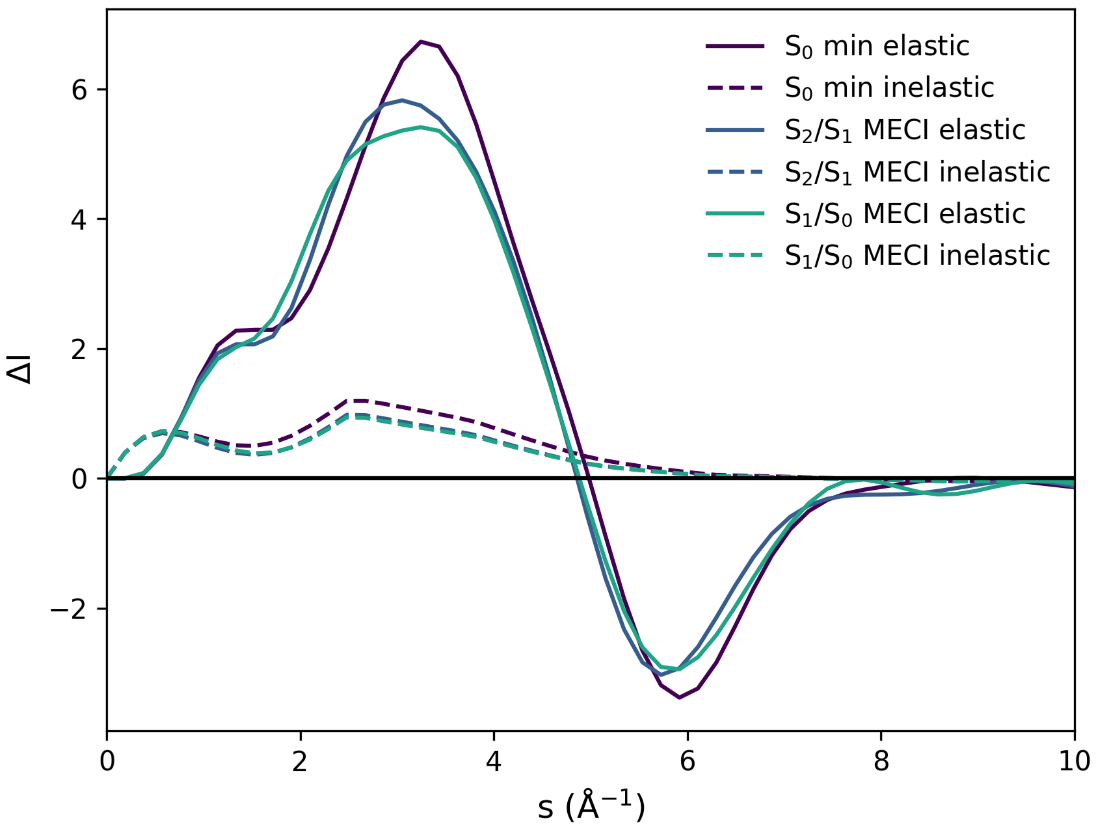

Figure S16: The difference (ΔI) of the IAM to AIS signals at the three optimized geometries of the LIIC separated into the elastic and inelastic components.

Figure SI S16 shows $\Delta I = I_{IAM} - I_{AIS}$ at the three optimized geometries in Fig. 1, separated into the elastic and inelastic components. Although errors are incurred in both the elastic and inelastic components, a larger absolute difference is seen in the elastic component. For the three geometries considered in Fig. SI S16, the changes in the inelastic scattering

are comparatively small (and, by design, absent from the IAM).

Clusters

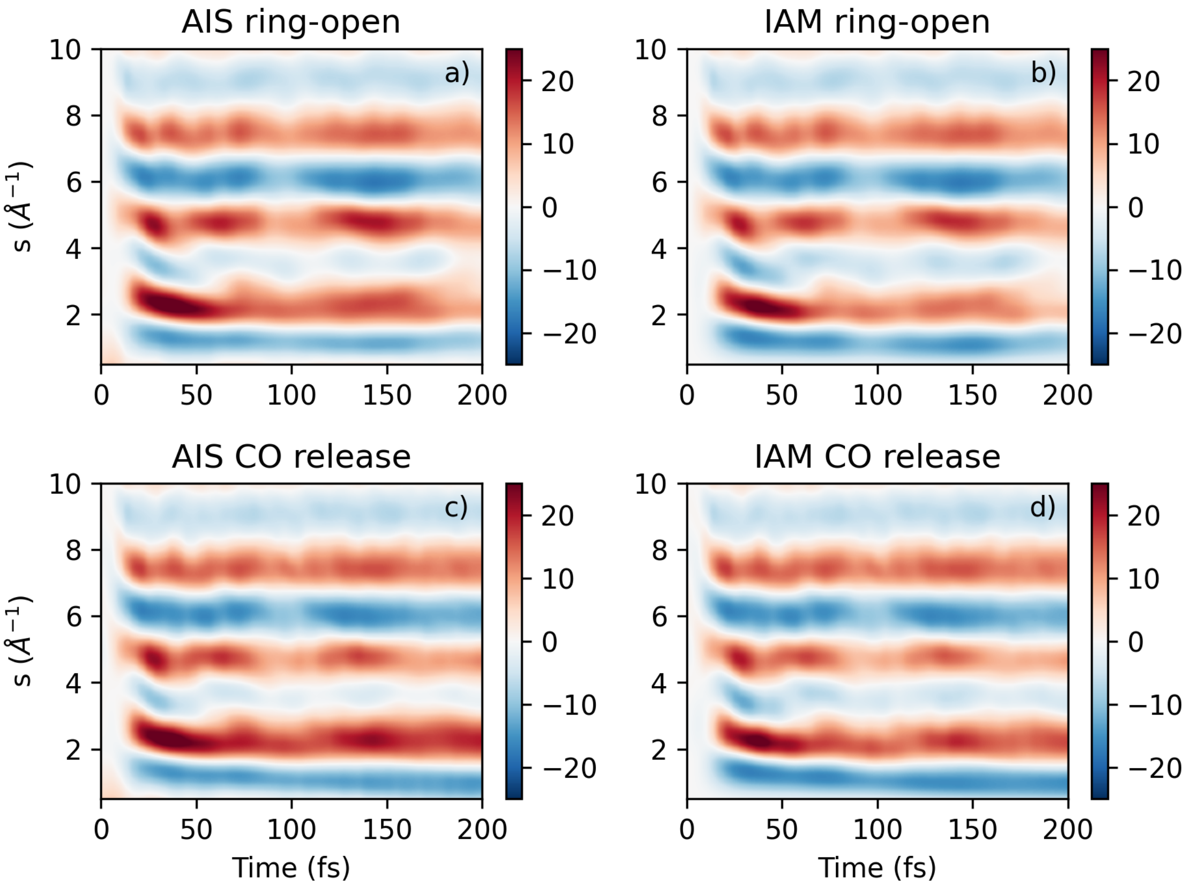

Figure S17: Isotropic UED signals for the ring-open and CO release reaction channel calculated using AIS and IAM.

Table S4: Ground state energies in atomic units and a breakdown into the nuclear, kinetic, one electron and two electron energies of γ-butyrolacetone for the active spaces employed in Figure SI Table 1.

Active space	Ground state energy	Nuclear energy	Kinetic energy	one electron energy	two electron energy
10,8	-304.806501407094	241.33019926	304.96788051	-893.93813080	347.80143014
8,7	-304.785567002714	241.33019926	304.94065012	-893.93905811	347.82329185
8,6	-304.771688571140	241.33019926	304.93467155	-894.01568520	347.91379737
6,5	-304.758472272395	241.33019926	304.91832421	-894.02727584	347.93860431
6,4	-304.736969783026	241.33019926	304.85774271	-894.00297563	347.93580659
4,3	-304.709196870943	241.33019926	305.09759911	-893.97016558	347.93076945

Relativistic correction

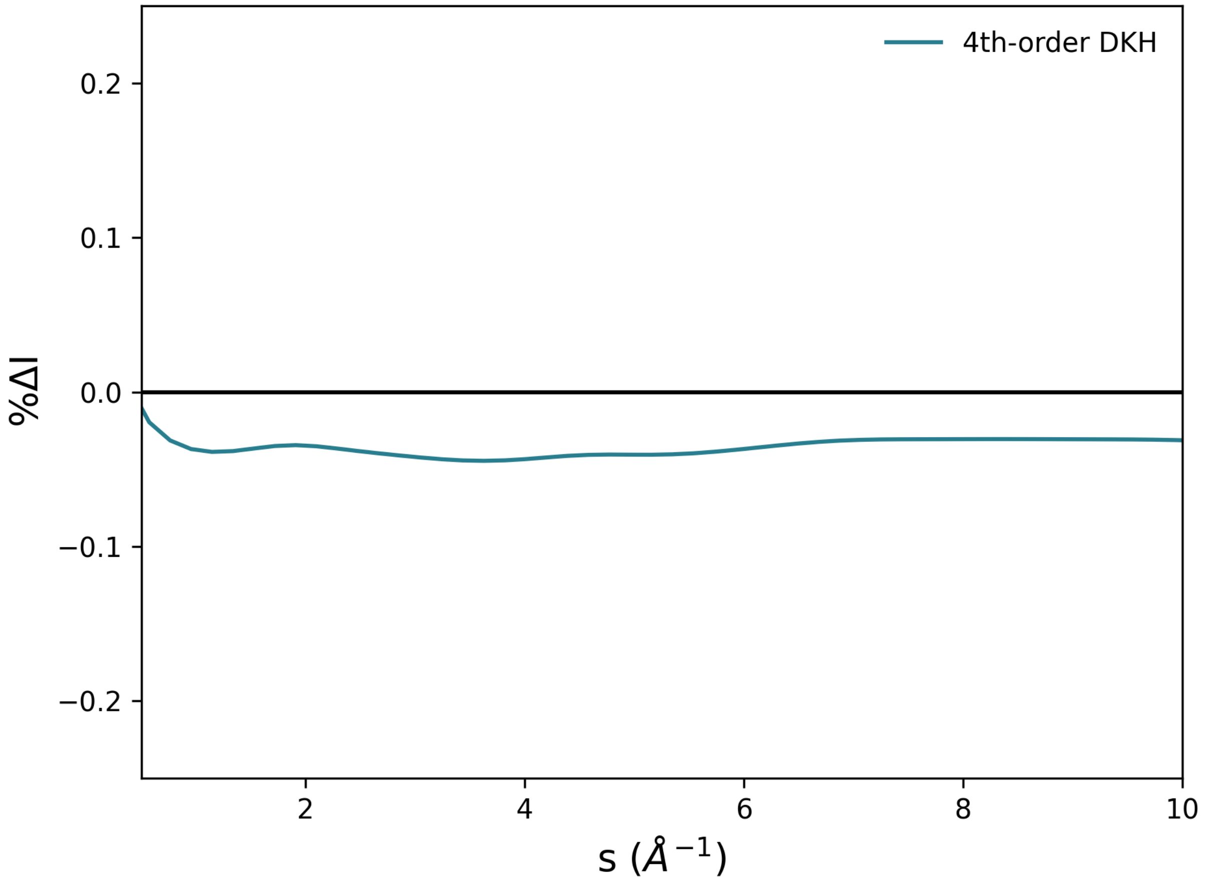

Figure S18: Scattering at the equilibrium geometry calculated using SA(3)-CASSCF(10,8)/cc-pVDZ as the reference against SA(3)-CASSCF(10,8)/cc-pVDZ with a 4th order DKH correction.

Fractional difference

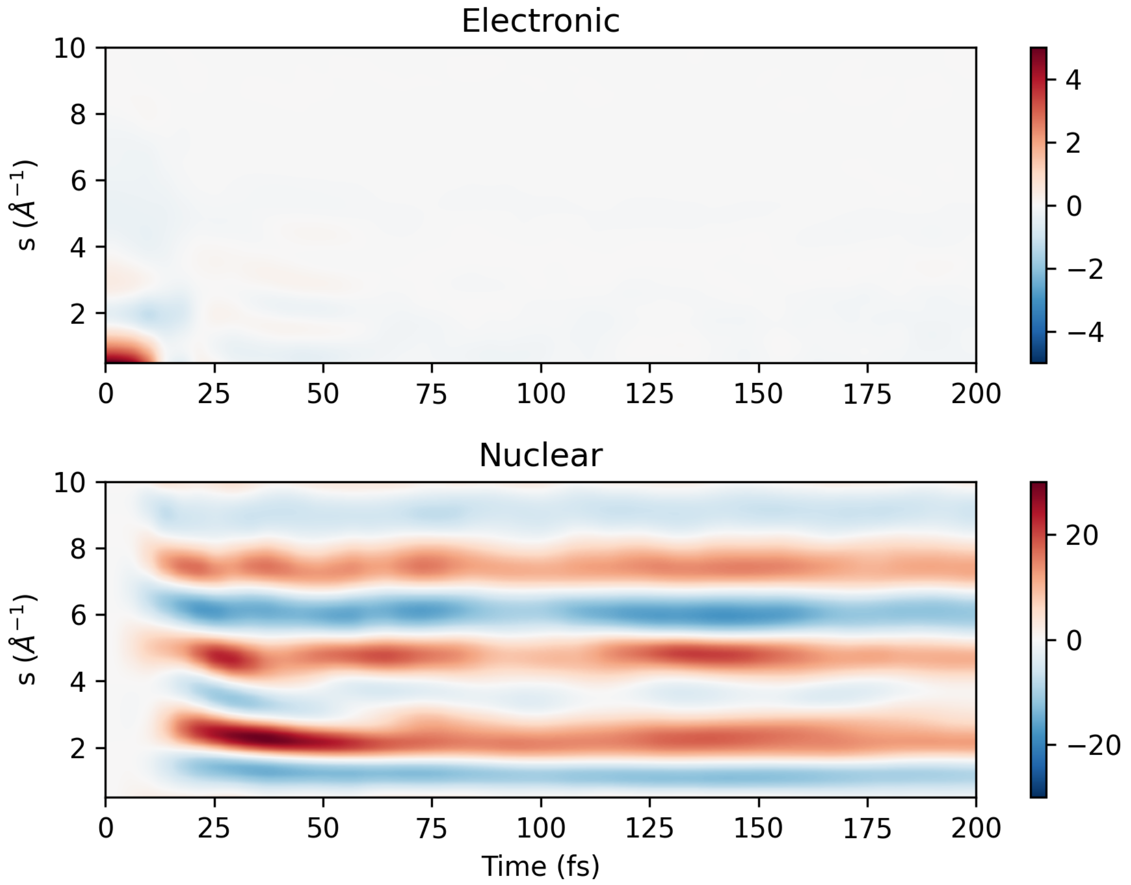

Figure S19: Decomposition of the UED signal into the electronic ($\%\Delta I_{\text{elec}}(s,t)$) and nuclear ($\%\Delta I_{\text{nucl}}(s,t)$) components obtained according to Ref. 5.

Convolution with a probe pulse

The full signal at $s = 1.3$ and 2.3 Å^{-1} is shown in Fig. SI S20 temporally convoluted by a Gaussian pulse with FWHM 130 fs. The convoluted signals are fitted to an error function in the region of $t = 0 - 200$ fs. This resulting in lifetimes (τ) for the peak at $s = 1.3$ Å^{-1} of 37.2 and 34.2 fs for AIS and IAM, respectively. Fitting to the peak at $s = 2.3$ Å^{-1} yields τ as 44.6 and 37.2 fs for AIS and IAM. This clearly indicates that the differences changes in intensity between AIS and IAM survive temporal convolution.

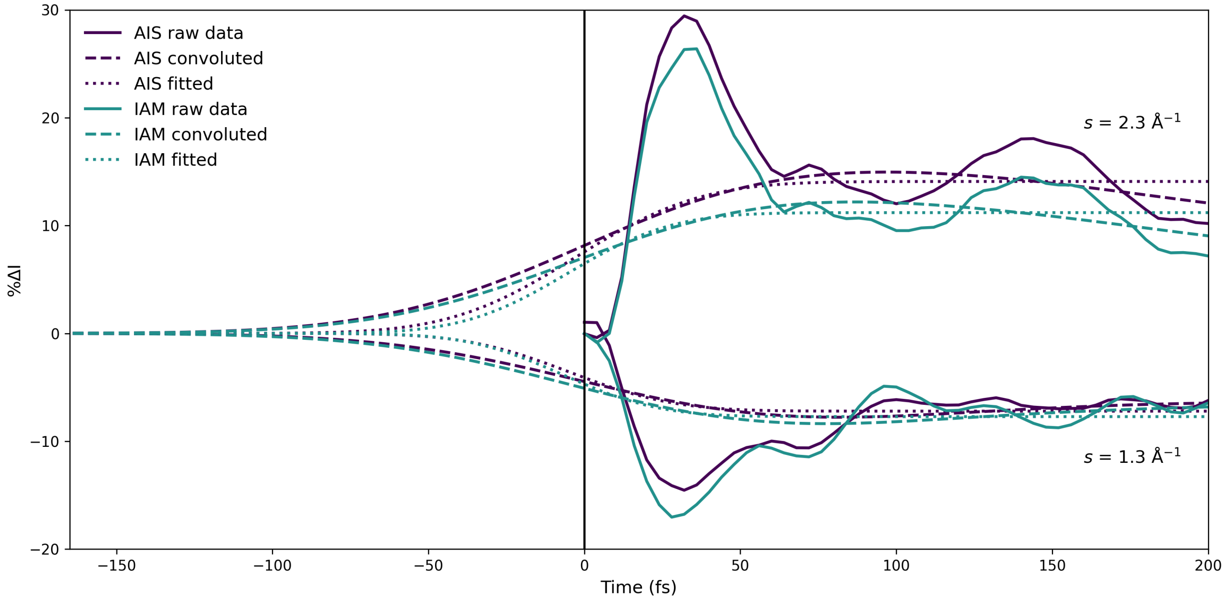

Figure S20: Signal intensity of two peaks ($s = 1,3,2.3$ Å^{-1}) shown in Fig. 6 for the full duration of the TSH simulations (solid lines). The signals are convoluted with a Gaussian pulse FWHM of 130 fs (dashed), then finally the convoluted data is fitted to the erf function of Eq. 7 (dotted).

References

(1) Schalk, O.; Galiana, J.; Geng, T.; Larsson, T. L.; Thomas, R. D.; Fdez Galván, I.; Hansson, T.; Vacher, M. Competition between ring-puckering and ring-opening excited state reactions exemplified on 5H-furan-2-one and derivatives. *J. Chem. Phys.* **2020**, *152*, 064301.

(2) Franzke, Y. J. et al. TURBOMOLE: Today and Tomorrow. *J. Chem. Theory Comput.* **2023**, *19*, 6859–6890.

(3) Köhn, A.; Hättig, C. Analytic gradients for excited states in the coupled-cluster model CC2 employing the resolution-of-the-identity approximation. *J. Chem. Phys.* **2003**, *119*, 5021–5036.

(4) Persico, M.; Granucci, G. An overview of nonadiabatic dynamics simulations methods,

with focus on the direct approach versus the fitting of potential energy surfaces. *Theor. Chem. Acc.* **2014**, *133*, 1526.

(5) Yong, H. et al. Observation of the molecular response to light upon photoexcitation. *Nat. Comm.* **2020**, *11*, 2157.
